# Supplementary material for: Qualitative interview study of antibiotics and self-management strategies for respiratory infections in primary care
Source: BMJ Open. 2017 Nov 26;7(11):e016903. doi: 10.1136/bmjopen-2017-016903 (PMC5719297; doi:10.1136/bmjopen-2017-016903)
Supplement: Supplementary file 1 [file bmjopen-2017-016903supp001.pdf]

Appendix 1

Introduction: Topics to be covered

The aim of this interview is to understand (1) the diagnosis and management of respiratory tract infections (RTIs) and your understanding of what has caused your infection (2) Patients' views on using paracetamol, ibuprofen and antibiotics.

If you could start at the beginning and tell me what led you/your child coming to see the doctor/nurse with your/your child's infection?

Prompts: Severity? Duration? Self-help- tried, if so what?

Previous experience of sore throat?
Previous experience of seeing doctor and getting treatment?
Family/friend/social support network?

We are interested in patient views on what causes RTI. Could you tell me what you think may have caused your illness or where you got it from?

Prompt: Bacteria? (If so, where from?) Virus? Weak system? Past/family history?

Could you please tell me if you have previously used paracetamol?

Frequency? Reason?

What are your views about taking paracetamol?

Prompt: Is taking full doses a problem or harmful in any way- e.g. is fever 'good'? Concerns?

Have you ever heard about problems with paracetamol?

e.g. poison; side effects

Do you remember the advice about paracetamol?

Prompt: Clear advice? Did you use it regularly rather than on demand? Did you use full doses?

If you stopped using it, why? (e.g. severity of symptoms settling, otherwise coping?)

Have you previously used ibuprofen? How do you feel about it?

Prompt: Is taking full doses a problem or harmful in any way- e.g. is fever 'good'?
Have you heard about problems with ibuprofen? (e.g. ulcers; side effects?)

Do you remember the advice about ibuprofen?
Did you use it regularly rather than on demand? Did you use full doses?
If you stopped using it, why? (e.g. severity of symptoms settling, otherwise coping?)

Have you previously used steam inhalation? How do you feel about it?

Prompt: offer an explanation if participant unsure.

What made you decide to use steam inhalation? Did you receive any advice?
How often did you use it? (e.g. regularly, sporadically).
If you stopped using it, why? (e.g. severity of symptoms settling, otherwise coping?)

If you haven't used steam inhalation, why? Is this something you would try in the future, if so why(not)?

Have you previously had antibiotics?
How did you feel about them?
Have you heard about problems with antibiotics (e.g. resistance; side effects?)

Do you remember the advice about antibiotics?
Were you advised to wait, if so, how long?
What was your experience of waiting?

Have you come across delayed prescribing before?
Prompt: Provide more information if participant unsure.

Outline the four common methods of delayed prescribing.

How did you feel about the method of delayed prescribing you were offered?
OR
How do you think you would feel about being offered delayed prescribing if you/your child consulted again?

If you started antibiotics, why? (e.g. severity of symptoms, general deterioration in well-being).

In your consultation with the doctor, how did you feel the doctor responded to your infection?"

Prompt: How did you feel about the consultation you had? Sympathetic? Enough information? Clear advice?

Were you given other self-help advice? If so, what did you think about it?

What other self-help measures did you use (e.g. tepid sponging?)

Conclusion

Are there any relevant issues we haven’t covered that you would like to mention?

Are there any questions you would like to ask me?
