# Supplementary material for: Qualitative interview study of antibiotics and self-management strategies for respiratory infections in primary care
Source: BMJ Open. 2017 Nov 26;7(11):e016903. doi: 10.1136/bmjopen-2017-016903 (PMC5719297; doi:10.1136/bmjopen-2017-016903)
Supplement: Author's manuscript [file bmjopen-2017-016903.draft_revisions.pdf]

BMJ Open is committed to open peer review. As part of this commitment we make the peer review history of every article we publish publicly available.

When an article is published we post the peer reviewers' comments and the authors' responses online. We also post the versions of the paper that were used during peer review. These are the versions that the peer review comments apply to.

The versions of the paper that follow are the versions that were submitted during the peer review process. They are not the versions of record or the final published versions. They should not be cited or distributed as the published version of this manuscript.

BMJ Open is an open access journal and the full, final, typeset and author-corrected version of record of the manuscript is available on our site with no access controls, subscription charges or pay-per-view fees (http://bmjopen.bmj.com).

If you have any questions on BMJ Open's open peer review process please email editorial.bmjopen@bmj.com

BMJ Open

‘Well I should have thought that was a bit of a lethal cocktail’: a nested qualitative interview study exploring the use of using self-management treatments and delayed antibiotic prescriptions for the management of respiratory infections (PIPS trial)

Journal:	*BMJ Open*
Manuscript ID	bmjopen-2017-016903
Article Type:	Research
Date Submitted by the Author:	17-Mar-2017

Complete List of Authors:	McDermott, Lisa; King's College London, Primary Care and Public Health Sciences<br>Leydon, Gerry; University of Southampton, Primary Care and Population Sciences<br>Halls, Amy; University of Southampton, Primary Care and Population Sciences<br>Kelly, Jo; University of Southampton, Primary Care and Population Sciences<br>Nagle, Amanda; University of Southampton, Primary Care and Population Sciences<br>White, Jennifer; University of Southampton, Primary Care and Population Sciences<br>Little, Paul; University of Southampton, Primary Care and Population Science;

**Primary Subject Heading**:	General practice / Family practice
Secondary Subject Heading:	Qualitative research
Keywords:	antibiotics, prescribing, respiratory tract infection, self-help

SCHOLARONE™
Manuscripts

'Well I should have thought that was a bit of a lethal cocktail': a nested qualitative interview study exploring the use of using self-management treatments and delayed antibiotic prescriptions for the management of respiratory infections (PIPS trial)

Authors:

McDermott L*±, Leydon GM**±, Halls A**, Kelly J**, Nagle A**, White J**, Little P**, on behalf of the PIPS investigators.

*Primary Care and Public Health Sciences, King's College, London

614, 6th Floor, Capital House, 42 Weston Street, London, SE1 3QD

**Primary Care and Population Sciences, Faculty of Medicine, University of Southampton

Aldermoor Health Centre, Aldermoor Close, Southampton, SO16 5ST

±These authors are to be regarded as Joint First Authors.

Corresponding author: GM Leydon gerry@soton.ac.uk

Key words: Antibiotics, prescribing, Respiratory Tract Infection, self-help

For peer review only

Abstract

Objective: To explore perceptions of illness, the decisions to consult, and the acceptability of delayed antibiotic prescriptions and self-help treatments for respiratory tract infections (RTIs).

Design: Qualitative semi-structured interview study.

Setting: UK Primary care.

Participants: 20 patients who had been participating in the 'PIPS' (Pragmatic Ibuprofen Paracetamol and Steam) trial in the South of England.

Method: Semi-structured, telephone interviews were conducted with participants to explore their experiences and views on various treatments for RTI.

Results: Participants had significant concerns about symptoms which were not serious and were mostly unaware of the natural history of RTIs, but were aware of the limitations of antibiotics and did not expect them with every consultation. Most viewed delayed prescriptions positively and had no strong preference over which technique is used to deliver the delayed antibiotic, but some patients received mixed messages, such as being told it was viral then being given an antibiotic, or were sceptical about the rationale. Participants disliked self-help treatments that involved taking medication and were particularly concerned about painkillers in combination. Steam inhalation was viewed as only moderately helpful for mild symptoms.

Conclusion: Delayed prescribing is acceptable no matter how the delay is operationalized, but explanation of the rationale is needed and care taken to minimise mixed messages. Better access is needed to good natural history information, and the signs and symptoms requiring or not requiring GP advice. Significant concerns about self-care medication are likely to need careful exploration in the consultation.

For peer review only

Article summary

Strengths and limitations of this study (related to methods)

1. The interviews permitted open exploration of delayed prescribing which is novel
2. A range of views were solicited and saturation was reached
3. Where there is existing literature this study resonates with it
4. Interviews are reports and not a window onto the actual events/experiences reported
5. Trial participation may have led to sample of participants particularly interested in this research and may not represent views of 'typical' patients.

For peer review only - http://bmjopen.bmj.com/site/about/guidelines.xhtml

Background

The overuse of antibiotics can contribute to the spread of resistant bacteria[1-2]. This problem is currently on the increase and has been identified by the World Health Organisation as a serious issue which must be addressed with urgency[3]. Despite evidence to suggest the limited benefit of antibiotics in the treatment of respiratory tract infections (RTI) in particular[4-5], antibiotics still account for >60% of all prescriptions in primary care[6] and primary care accounts for 80% of human antibiotic consumption[7]. The delayed prescribing of antibiotics is a technique which may help to reduce unnecessary prescribing in primary care[8]. The method is recommended by the NICE guidelines[6] and involves a prescription being issued by the GP for patient use at a later date if symptoms do not improve. This can be delivered using a number of techniques which include four common methods – providing an unaltered prescription to the patient, providing a post-dated prescription, a telephone call to the practice in order to issue a prescription, and the prescription being left at the surgery reception for the patient to collect if they feel it necessary. In addition, the use of self-help over-the-counter medications and techniques can also be used to help relieve symptoms of RTI without the use of antibiotics: these include the use of paracetamol, ibuprofen and steam inhalation[9-11].

The PIPS (Pragmatic Ibuprofen Paracetamol and Steam) trial aimed to assess the effectiveness of four common methods of delayed prescribing outlined above, and the use of analgesics (paracetamol; ibuprofen or both) and steam inhalation for RTI. This paper reports on a qualitative study nested within the PIPS trial in order to determine the feasibility and acceptability of these management strategies from the perspective of participating patients and to investigate factors that are currently influencing their decisions to consult a GP.

Methods

Participants and procedure

Participants were recruited from a sample of patients presenting with RTI participating in the PIPS trial who had consented to being contacted for an interview. The participants were recruited from areas across Hampshire. Interview participants were purposively sampled to include a maximum possible variety of trial stage (from beginning to end of trial), trial arm (e.g. delayed prescribing method), age and gender. Participants were recruited by telephone.

For peer review only

Interviews

Trained interviewers (LM, AN, JW) conducted telephone interviews, with each lasting approximately half an hour. All interviews were audio–recorded and transcribed verbatim. Qualitative interviews provided the best method for gathering insights into participants' individual views about and experiences of treatments for RTI. A semi-structured interview guide (Appendix 1) included key topic areas while also providing flexibility to explore unanticipated issues.

Analysis

Inductive thematic analysis[12] was conducted on all transcripts to determine factors which influence patients' decision to consult a GP or use an alternative treatment for RTI. Following immersion in the transcripts, familiarisation was achieved and patterns and prominent themes which consistently occurred in the data were identified and labelled with codes. Each code label referred directly to the operationalisation of the theme content. A label and full descriptive definition was then provided for each theme. Codes and definitions were refined during a continuing process, which involved themes being linked, grouped, moved, re-labelled, added and removed to produce a set of themes and subthemes and a coding manual, which adequately fitted and thoroughly explained the data. The coding was iteratively developed across authors (led by LM and GL) and adjustments made where appropriate based on discussion.

Findings

Participants:

Twenty people participated, with ages ranging from 18-74 (mean age 51), and 70% (N=14) were female. In addition approximately 50% (N=11) of participants had completed the trial at the time of interview. All patients had experienced at least two or three different treatment options for RTI either as part of the trial or in the past.

Themes:

Thematic analysis identified a total of five key themes relating to patients' views of different management approaches, and factors which may influence a patient's decision to use a self-

For peer review only

help treatment (paracetamol/ibuprofen/steam inhalation) or consult with a GP for the treatment of a RTI.

Table 1: Themes identified in analysis

Themes	Sub-themes	
**1. Perceptions of illness**		-Duration of symptoms<br>-Perceived signs of severity<br>-Quality of life impact
**2. Advice from others**		-Acceptance of GP advice<br>-Consideration of alternative advice
**3. Perceptions of antibiotics**		-Antibiotics have unpleasant side-effects<br>-Concerns and misunderstandings about resistance<br>-Antibiotics needed for specific cases
**4. Perceptions of self-help treatment**		-Treatment duration short and irregular<br>-Experience of effective treatment
	**Paracetamol and ibuprofen**	-Apprehension of combination medication<br>-General dislike of medication<br>-Concerns of medication side-effects<br>-Medication acceptable for pain relief
	**Steam inhalation as a limited technique**	-Only beneficial if not severe<br>-Provides short term relief<br>-Relieves but not cures
**5. Perceptions of delayed prescribing**		-Viewed as a positive option<br>-Acceptance of GP recommended method<br>-Confusion of delayed prescribing role.

In the following sections we summarise themes 1 and 2 for context before reporting in depth on themes 3-5, describing each in turn, using exemplary quotations for illustrative purposes (participant number is shown in parentheses).

Perceptions of severity, and advice from others

Participants' views on the severity of their condition related to the evaluation of the duration of symptoms. Participants signalled that they were mostly unaware of the natural history, and

For peer review only - http://bmjopen.bmj.com/site/about/guidelines.xhtml

For peer review only

specific symptoms (such as breathing difficulty) were often highlighted as indicators that their condition might be severe.

> *"I think it was because it was - I could feel or could hear a creaking or croaking in the chest cavity…So that said to me, right we need to go and get something to deal with it."(PL7)*

Participants reported making decisions on how to treat their condition based on advice from both GPs and alternative professional and lay sources which included pharmacists, family members and the media.

> *"I do the Karvol inhalation, my patent remedy…. That's an old one from my mother, so it's a long, long time ago, but….I think that'd be the best treatment"(P03)*

3. Perceptions of antibiotics

A patient's decision on how to treat their condition was reported as being influenced by their perceptions of antibiotics, relating to beliefs about unpleasant side effects, concerns about resistance, and the belief that antibiotics are required for specific conditions and situations. Patients who described unpleasant side-effects associated with the use of antibiotics tended to report reluctance to take them, unless absolutely necessary.

> *"I don't like taking antibiotics because, after all, whatever it does to the system in relation to the bowel side of it, it destroys all the bacteria there anyhow so it's better to, if you can get away without taking them, the better"(PL08)*

In line with existing literature that identified people may believe the body to be resistant rather than the bacteria[13], many participants reported concerns about antibiotic resistance, which influenced their decision on how to best treat their condition and symptoms. Participants reported the belief that antibiotics were necessary in some specific situations and circumstances, such as patients with co-morbidities.

> *"Well in my own particular case … I almost feel that if I do go the doctor's with a nasty cough or some sort of … you know, nasal blockage or ear ache, I almost*

For peer review only - http://bmjopen.bmj.com/site/about/guidelines.xhtml

thought I ought to be given antibiotics as a matter of course because of my asthma" (PL7)

4. Perceptions of self-help treatment

Participants' perceptions surrounding the potential benefits and limitations of self-help treatments for RTI influenced their decision to try these or visit their GP. They reported that in general self-help treatments were used for short periods of time at irregular intervals, in contrast with the likely prescribed management approach offered by a GP to take pain relief at regular intervals.

"My GP did say that managed pain is better, so take it regularly, but I tend not to do that; perhaps have some in the morning and if I feel really ropey in the afternoon, but then definitely in the evening." (PL8)

Participants reported being apprehensive about taking a combination of ibuprofen and paracetamol together. The combination of medication was construed by some as somehow 'risky' when compared to taking a single type of medication.

"Well I should have thought that was a bit of a lethal cocktail together but I don't know; if it was up to me I wouldn't want to do that."(PL08)

In general, participants reported a dislike for self-help treatments which involved taking medication. However, it is interesting to note that despite views of disliking these medications, participants were more likely to use them if the GP advised it, and would be less likely to try these without such advice. Interviewees reported concerns and worries relating to possible side effects which taking paracetamol or ibuprofen may cause.

"Yes, isn't there a problem with that [ibuprofen] in relation to blood? Yes, there's something I've heard about ibuprofen, that particular medication, which I didn't think was too good...I would not want to take it in that sense."(PL01)

For peer review only

Despite concerns relating to taking paracetamol or ibuprofen for the treatment of RTI, participants reported that they were happy to take these for the use of pain relief. This was a familiar usage and appeared to be considered as more acceptable than using them for an RTI. Most reported steam inhalation could relieve symptoms to a degree, however it was perceived as only providing short-term relief from symptoms and that it would not assist in curing the underlying condition. Therefore if symptoms were considered as serious, using steam inhalation was not viewed as a potential overall treatment option.

> *"I don't think that they actually cure me, but I think they give me a little bit of relief from congestion." (PL09)*

5. Perceptions of delayed prescribing

Delayed prescribing was generally viewed as a positive technique and patients reported being happy to accept whichever method of delayed prescribing the GP recommended. However, there was some confusion reported as to the role of delayed prescribing. It was generally viewed by participants as a positive technique which could give them reassurance of having access to a prescription just in case. The main benefit was seen as the patient having some decision in their own treatment while preventing them from unnecessarily taking antibiotics.

> *"I don't mind delayed prescribing. I mean, I would rather have them and know that I've got them and, yes, if I don't need them, then I wouldn't take them, but I like the safety [of a delayed prescription], knowing that I've got them, because more often than not, it doesn't go away" (P05)*

Although happy to accept delayed prescribing as a treatment option, many participants were confused as to the purpose of the technique. Some reported confusion because they had been told their condition was viral yet had still been issued a delayed prescription, indicating the rationale for delayed prescribing may need to be clearer.

> *"…either you have or haven't got an infection that either does or doesn't need treating. And if it needs treating it needs treating now, if not it doesn't" (PA7)*

Some expressed the view that the technique might be driven by economic reasons.

For peer review only - http://bmjopen.bmj.com/site/about/guidelines.xhtml

"I am more and more wondering if medication is being withheld because of expenses…surgeries have to make a living, they have to work within a budget…" (PA3)

Discussion

The study identified five key themes relating to factors that influence patients' decisions to consult a GP or use a self-help treatment for a RTI. Findings are discussed in relation to the most prevalent and influential themes outlined by participants.

Main findings

Overall, the findings suggest participants were aware of the limitations of antibiotics and do not wish to receive them every time they consult a GP. There was some confusion about the rationale for delayed prescribing with a particular risk of mixed messages, whereby on the one hand some expressed the conflict between being told their condition did not warrant immediate treatment because it may not be a bacterial infection, whilst on the other hand it might at some point require antibiotic treatment. This is a complicated message for GPs to deliver and for patients to receive, interpret and understand. Some suspected that delayed prescribing was primarily driven by economic decisions and the need to cut spending, but most participants viewed delayed prescriptions positively, suggesting they were happy to accept their GP's recommendation. That said, what cannot be emphasised enough is the clear need for GPs and their patients to negotiate the delayed disposal and to render transparent the rationale for a delay.

Participants expressed concerns over self-help that involved taking medication, particularly painkillers in combination for the treatment of RTI. When patients did take medication some indicated irregular use of them, which contrasts with a need for regular and continued dosing, sometimes when symptoms appear to have reduced. Just taking 'drugs' when they feel they need to is intuitively logical and may signal a need for clearer information that people could get better and feel better more quickly if they take medication as recommended. Indeed, it seemed overall, despite reservations, participants conveyed an overall stance of a willingness to accept advice to use painkillers if recommended by their GP. Some interviewees seemed to require authorisation from their GP to try over-the-counter remedies: this may link to a

For peer review only - http://bmjopen.bmj.com/site/about/guidelines.xhtml

misconception that analgesics do not help for respiratory symptoms. Whilst steam inhalation was viewed as an acceptable treatment participants generally described this method as providing short-term relief from non-severe symptoms. The decision to consult a GP was initially influenced by patient perceptions of the natural history, a variety of personal indicators, and beliefs largely unrelated to documented natural history indices of severity.

Comparison with existing literature

Participants reported being aware of the limitations of antibiotics and not expecting to receive them every time they went to the GP with a RTI which supports previous research, and often contrasts with GP perceptions of high levels of patient pressure to prescribe[8]. However, the self-selected nature of the sample interviewed is likely to mean they were more open to exploring alternatives to antibiotics[14]. There is still considerable room for a better understanding of the limited benefit from antibiotics given the evidence that beliefs in the effectiveness of antibiotics are still strong[15-16]. There is also room to acknowledge the views held by some patients to ensure potential misunderstandings are managed well when a no-prescribing disposal is selected. For example, whilst not common, some interview accounts suggested that not prescribing antibiotics can be understood (at least in part) to be a resource saving strategy rather than a clinically driven one. Tonkin-Crine et al.[17] also found that older adults may hold such a view. In addition, the perception of a delayed script as potentially contradictory also needs to be tackled when a prescribing decision is negotiated. Patients reported high levels of concordance with GP advice across all treatment options, including all methods of delayed prescribing. Previous studies suggest high patient compliance/concordance is strongly associated with doctor-patient agreement, which is facilitated by patient understanding of treatment benefits[18]. Although not discussed in detail, data suggested participants would have been happy to accept a delayed prescription: these perceptions were supported by the quantitative satisfaction data from the trial[15]. Previous research identified patient satisfaction with delayed prescribing[19], but also a subgroup of patients who had expected antibiotics and were disappointed when they did not receive a prescription for immediate use. The current study in contrast did not find a group of patients who were disappointed at not receiving antibiotics. Speculatively, this may, in part, be associated with societal changes and the extensive publicity about antibiotic resistance. However, this also may reflect selection bias since trial patients may represent a sub-group who are interested in research, have more awareness of issues surrounding antibiotic

For peer review only

prescribing, and are more open to trying different approaches and therefore happier to accept new advice or techniques. Furthermore, since all participants were taking part in a trial it is likely that GPs provided a detailed explanation of their treatment decision, using structured advice sheets, which is unlikely to happen in routine practice [20].

Implications for clinical practice

Overall, participants reported delayed prescribing to be an acceptable treatment option. Previous research[21] has suggested that patient understanding of the rationale for a particular treatment can increase patient satisfaction. In this study delayed prescribing was delivered using structured advice sheets detailing the expected natural history; benefits/disbenefits of antibiotics; how long to delay; and safety netting/symptom advice. The findings suggest good concordance and high satisfaction should accompany a consistent and thorough explanation of the approach. Participants did not display a strong preference of any particular method of delayed prescribing, which suggests any approach is likely to be acceptable in practice, as long as a clear explanation is provided. This is similar to the information GPs were instructed to provide patients with as part of the trial protocol in this study.

Findings suggest participants were concerned about using painkillers as a treatment method for RTI. They reported concerns relating to potential hazards of combining analgesics, which links with existing evidence of low analgesic concordance rates in patients with RTI[22]. However, interviewees did report that they thought paracetamol and ibuprofen were, at least in isolation, acceptable treatments for pain relief. This finding suggests that analgesics can be viewed as feasible treatment options for patients, but education and explanation of the justification and possible benefits/dis-benefits for these medications need to be shared with patients. The results of the main trial suggest that advice to consume regular ibuprofen either alone or in combination may be associated with worse outcomes and hence a preference for paracetamol would be the recommended approach in future[15].

Patients' decisions to consult a GP were often related to perceived indicators of illness severity which were more often incorrect than correct, and beliefs relating to whether they thought they had caught the same condition as other people they knew. These findings are consistent with Leventhal's (1984) model of illness representations[23] which suggests that patients' beliefs relating to the cause, identity, duration and controllability of an illness are all

For peer review only - http://bmjopen.bmj.com/site/about/guidelines.xhtml

likely to influence the way in which they respond to the condition (e.g. consulting a GP). The GRACE INTRO European study used this model to develop a patient booklet and findings indicated it was well received by patients and gave them new information about illness duration and how to self care[17,24]. As most of the indicators that patients used to determine illness severity may not warrant a GP consultation, it suggests a need to review the clarity of patient information (e.g. providing clear examples of symptoms and illness duration that would ordinarily merit a visit to the GP, and those which are not a cause for worry). The promotion of this information within the community, as well as dealing with the common misconception that antibiotic medication may help coughs and colds[16] could help to reduce unnecessary GP consultations in the future.

Strengths and limitations

One of the key strengths of the study is that all patients interviewed had experienced at least two or three different treatment options for RTI either as part of the trial or in the past. This included antibiotics, delayed prescribing, paracetamol, ibuprofen, and steam inhalation. Therefore, the interviews provided opportunity for discussion of the various treatment options with a targeted information-rich sample. This strengthens the findings and allows stronger conclusions to be drawn.

However, the sample of participants who took part in the interviews may have limited the scope of the findings somewhat, as they had all participated in the PIPS trial. This may have led to a sample of participants who were particularly interested in research of this nature and may not have represented views held by 'typical' patients, particularly views on antibiotic resistance.

Conclusion

The findings suggest that delayed prescribing appears to be an acceptable technique, as long as such a disposal is described well to optimise patient understanding of the rationale. Increased patient education relating to the safety and justification of using paracetamol for the treatment of RTI is likely to increase the acceptability of analgesics. In addition, enhanced approaches to sharing information with patients- relating to the natural history of illness, the appropriate signs and symptoms requiring GP advice, and those symptoms and signs which do not require urgent attention – could help reduce unnecessary consultations.

For peer review only

Acknowledgements

We are grateful to all the general practitioners and practice nurses and particularly the patients who gave their time to this study.

PIPS Investigators

University of Southampton: Paul Little, Ian Williamson, Mike Moore, Mark Mullee, Jo Kelly, Julie Hooper, Lisa McDermott, Geraldine Leydon, Ben Holdstock-Brown*, Amanda Nagle*, Jennifer White* (*medical students at the time); Samantha Hall, Patient and Public (PPI) representative.

Author contribution

GM Leydon led the qualitative work as part of the PIPs trial. P Little secured research funding/and acted as overall PI of the PIPs trial. J Kelly facilitated recruitment. L McDermott, A Nagle, J White, and B Holdstock-Brown collected interview data. All authors were involved in/commented on data analysis (led by G Leydon and L McDermott). A Halls developed the manuscript, to which all authors contributed.

Funding statement

This article presents independent research funded by the National Institute for Health Research (NIHR) under its Programme Grants for Applied Research programme (grant ref No RP-PG-0407-10098). The views expressed are those of the author(s) and not necessarily those of the NHS, the NIHR or the department of Health.

Competing interests statement

There are no competing interests to report.

Ethical approval

The study was approved by Southampton and Southwest MREC number 06/Q1702/154 and all participants gave informed consent.

Data sharing

No additional data available.

For peer review only

References

1. Dagan R, Leibovitz E, Greenberg D, Yagupsky P, Fliss DM, Leiberman A. Dynamics of pneumococcal nasopharyngeal colonization during the first days of antibiotic treatment in pediatric patients. *J Ped Infect Dis* 1998; 17:880-5.
2. Costelloe C, Metcalfe C, Lovering A, Mant D, Hay A. Effect of antibiotic prescribing in primary care on antimicrobial resistance in individual patients: Systematic review and meta-analysis. *BMJ* 2010;340:c.2096.
3. World Health Organisation. The evolving threat of antimicrobial resistance: Options for action. WHO; 2012.
4. Little P, Watson L, Morgan S, Williamson I. Antibiotic prescribing and admissions with major suppurative complications of respiratory tract infections: a data linkage study. *Br J Gen Pract* 2002;52(476):187-90.
5. Macfarlane J, Holmes W, Gard P, Thornhill D, Macfarlane R, Hubbard R. Reducing antibiotic use for acute bronchitis in primary care: blinded, randomised controlled trial of patient information leaflet. *BMJ* 2002; 324:91-4.
6. NICE Guideline Development Group. Respiratory tract infections – antibiotic prescribing: Prescribing of antibiotics for self-limiting respiratory tract infections in adults and children in primary care. London: NICE; 2008
7. ESPAUR report Public Health England. English surveillance programme for antimicrobial utilisation and resistance (ESPAUR) 2010-2014. London: Public Health England; 2015.
8. Little P, Rumsby K. Kelly J, Watson L, Moore M, Warner G, et al. Information leaflet and antibiotic prescribing strategies for acute lower respiratory tract infection: a randomised controlled trial. *JAMA* 2005;293:3029-35.
9. Hollinghurst S, Redmond N, Costelloe C, Montgomery A, Fletcher M, Peters TJ, Hay AD. Paracetamol plus ibuprofen for the treatment of fever in children (PITCH). *BMJ* 2008;337:a1490.
10. Singh M, Heated, humidified air for the common cold. *Cochrane Library* 2004;2:CD001728.
11. Burke P, Bain J, Robinson, D, Dunleavey J. Acute red ear in children: controlled trial

For peer review only

of non-antibiotic treatment in general practice. *BMJ* 1991;303:558-562.

12. Braun V, and Clarke V, Using thematic analysis in psychology. *Qual Res in Psych* 2006; 3:77-101.
13. Brookes-Howell L, Elwyn G, Hood K, Wood F, Cooper L, Goossens H, Ieven M, Butler CC. 'The body gets used to them': patients' interpretations of antibiotic resistance and the implications for containment strategies *J Gen Intern Med* 20120;27(7):766-72.
14. McNulty CAM, Nichols T, French DP, Joshi P, Butler CC. 'Expectations for consultations and antibiotics for respiratory tract infection in primary care: the RTI clinical iceberg *Brit J Gen Pract* 2013;63(612):e.429-e.436.
15. Little P, Moore M, Kelly J et al. Ibuprofen, paracetamol, and steam for patients with respiratory tract infections in primary care: pragmatic randomised factorial trial *BMJ* 2013;347:f6041.
16. McNulty CAM, Boyle P, Nichols T, Clappison P, and Davey P. The public's attitudes to and compliance with antibiotics. *J Antimicrob Chemo* 2007; 60 (suppl 1):i63-i68.
17. Tonkin-Crine S, Anthierens S, et al. 'Exploring patients' views of primary care consultations with contrasting interventions for acute cough: a six-country European qualitative study, npj *Primary Care Respiratory Medicine* 2014;24:14026.
18. Kerse N, Buetow S, Mainous AG, Young, G, Coster G, Arroll B. 'Physician-patient relationship and medication compliance: A primary care investigation. *Ann Fam Med* 2004;2:455-461.
19. Arroll BA, Goodyear-Smith F, Thomas DR, Kerse N. Delayed antibiotic prescriptions: what are the experiences and attitudes of physicians and patients? *J Fam Pract* 2002;51;954-9.
20. Little P, Moore M, Kelly J, Williamson I, Leydon G, McDermott L, Mullee M, Stuart B. Delayed antibiotic prescribing strategies for respiratory tract infections in primary care: pragmatic, factorial, randomised controlled trial. *BMJ* 2014; 14:348
21. Francis NA, Phillips R, Wood F, Hood K, Simpson S, Butler CC. Parents' and clinicians' views of an interactive booklet about respiratory tract infections in children: a qualitative process evaluation of the EQUIP randomised controlled trial. *BMC Family Practice* 2013;14:182.
22. Little P, Gould C, Williamson I, Moore M, Warner G, Dunleavey J. A pragmatic randomised controlled trial of two prescribing strategies for acute otitis media. BMJ 2001;332:336-42.

For peer review only

23. Leventhal H, Nerenz DR, Steel DJ. Illness representations and coping with health threats. In: Baum A, Taylor SE, Singer JK, editors. Handbook of Psychology and Health: Social psychological aspects of health. Hillsdale, NJ: Earlbaum, 1984.
24. Yardley L, Douglas E, Athierens S, Tonkin-Crine S, O'Reilly G, Stuart B. et al. Evaluation of a web-based intervention to reduce antibiotic prescribing for LRTI in six European countries: quantitative process analysis of the GRACE/INTRO randomised controlled trial *Implementation Science* 2013;8:134

For peer review only

Appendix 1

Summary list of topics explored during interview

Part one: Experiences of RTI

Questions asked in this section covered the participants' experiences of RTI, experiences of visiting the GP, and advice given in the index consultation.

Part two: Views and experience of treatment of RTIs

Questions asked in this section covered participant views and experiences of different methods of treating RTIs, including paracetamol, ibuprofen, steam and antibiotics; understandings of antibiotics, their use and side effects; advice received (if any) from the GP, and views on different methods for prescribing antibiotics.

For peer review only

COREQ (COnsolidated criteria for REporting Qualitative research) Checklist

A checklist of items that should be included in reports of qualitative research. You must report the page number in your manuscript where you consider each of the items listed in this checklist. If you have not included this information, either revise your manuscript accordingly before submitting or note N/A.

Topic	Item No.	Guide Questions/Description	Reported on Page No.
**Domain 1: Research team and reflexivity**			
Personal characteristics			
Interviewer/facilitator	1	Which author/s conducted the interview or focus group?	4
Credentials	2	What were the researcher's credentials? E.g. PhD, MD	
Occupation	3	What was their occupation at the time of the study?	
Gender	4	Was the researcher male or female?	
Experience and training	5	What experience or training did the researcher have?	4
Relationship with participants			
Relationship established	6	Was a relationship established prior to study commencement?	
Participant knowledge of the interviewer	7	What did the participants know about the researcher? e.g. personal goals, reasons for doing the research	
Interviewer characteristics	8	What characteristics were reported about the inter viewer/facilitator? e.g. Bias, assumptions, reasons and interests in the research topic	
**Domain 2: Study design**			
Theoretical framework			
Methodological orientation and Theory	9	What methodological orientation was stated to underpin the study? e.g. grounded theory, discourse analysis, ethnography, phenomenology, content analysis	
Participant selection			
Sampling	10	How were participants selected? e.g. purposive, convenience, consecutive, snowball	3
Method of approach	11	How were participants approached? e.g. face-to-face, telephone, mail, email	3
Sample size	12	How many participants were in the study?	4
Non-participation	13	How many people refused to participate or dropped out? Reasons?	
Setting			
Setting of data collection	14	Where was the data collected? e.g. home, clinic, workplace	
Presence of non-participants	15	Was anyone else present besides the participants and researchers?	
Description of sample	16	What are the important characteristics of the sample? e.g. demographic data, date	4
Data collection			
Interview guide	17	Were questions, prompts, guides provided by the authors? Was it pilot tested?	4 and appendi
Repeat interviews	18	Were repeat inter views carried out? If yes, how many?	
Audio/visual recording	19	Did the research use audio or visual recording to collect the data?	
Field notes	20	Were field notes made during and/or after the inter view or focus group?	
Duration	21	What was the duration of the inter views or focus group?	3
Data saturation	22	Was data saturation discussed?	
Transcripts returned	23	Were transcripts returned to participants for comment and/or	

For peer review only

Topic	Item No.	Guide Questions/Description	Reported on Page No.
		correction?	
**Domain 3: analysis and findings**			
Data analysis			
Number of data coders	24	How many data coders coded the data?	4
Description of the coding tree	25	Did authors provide a description of the coding tree?	
Derivation of themes	26	Were themes identified in advance or derived from the data?	4
Software	27	What software, if applicable, was used to manage the data?	
Participant checking	28	Did participants provide feedback on the findings?	
Reporting			
Quotations presented	29	Were participant quotations presented to illustrate the themes/findings? Was each quotation identified? e.g. participant number	5 onwards
Data and findings consistent	30	Was there consistency between the data presented and the findings?	5
Clarity of major themes	31	Were major themes clearly presented in the findings?	5
Clarity of minor themes	32	Is there a description of diverse cases or discussion of minor themes?	

Developed from: Tong A, Sainsbury P, Craig J. Consolidated criteria for reporting qualitative research (COREQ): a 32-item checklist for interviews and focus groups. *International Journal for Quality in Health Care*. 2007. Volume 19, Number 6: pp. 349 – 357

Once you have completed this checklist, please save a copy and upload it as part of your submission. DO NOT include this checklist as part of the main manuscript document. It must be uploaded as a separate file.

For peer review only

BMJ Open

A qualitative interview study of antibiotics and self-management strategies for respiratory infections in primary care.

Journal:	*BMJ Open*
Manuscript ID	bmjopen-2017-016903.R1
Article Type:	Research
Date Submitted by the Author:	05-Jun-2017
Complete List of Authors:	McDermott, Lisa; King's College London, Primary Care and Public Health Sciences<br>Leydon, Gerry; University of Southampton, Primary Care and Population Sciences<br>Halls, Amy; University of Southampton, Primary Care and Population Sciences<br>Kelly, Jo; University of Southampton, Primary Care and Population Sciences<br>Nagle, Amanda; University of Southampton, Primary Care and Population Sciences<br>White, Jennifer; University of Southampton, Primary Care and Population Sciences<br>Little, Paul; University of Southampton, Primary Care and Population Science;
**Primary Subject Heading**:	General practice / Family practice
Secondary Subject Heading:	Qualitative research
Keywords:	antibiotics, prescribing, respiratory tract infection, self-help

SCHOLARONE™
Manuscripts

A qualitative interview study of antibiotics and self-management strategies for respiratory infections in primary care.

Authors:

McDermott L*±, Leydon GM**±, Halls A**, Kelly J**, Nagle A**, White J**, Little P**, on behalf of the PIPS investigators.

*Primary Care and Public Health Sciences, King's College, London
614, 6th Floor, Capital House, 42 Weston Street, London, SE1 3QD

**Primary Care and Population Sciences, Faculty of Medicine, University of Southampton
Aldermoor Health Centre, Aldermoor Close, Southampton, SO16 5ST

±These authors are to be regarded as Joint First Authors.

Corresponding author: GM Leydon gerry@soton.ac.uk

Key words: Antibiotics, prescribing, Respiratory Tract Infection, self-help

For peer review only

Abstract

Objective: To explore perceptions of illness, the decisions to consult, and the acceptability of delayed antibiotic prescriptions and self-help treatments for respiratory tract infections (RTIs).

Design: Qualitative semi-structured interview study.

Setting: UK Primary care.

Participants: 20 adult patients who had been participating in the 'PIPS' (Pragmatic Ibuprofen Paracetamol and Steam) trial in the South of England.

Method: Semi-structured, telephone interviews were conducted with participants to explore their experiences and views on various treatments for RTI.

Results: Participants had concerns about symptoms which were not clinically serious and were mostly unaware of the natural history of RTIs, but were aware of the limitations of antibiotics and did not expect them with every consultation. Most viewed delayed prescriptions positively and had no strong preference over which technique is used to deliver the delayed antibiotic, but some patients received mixed messages, such as being told their infection was viral then being given an antibiotic, or were sceptical about the rationale. Participants disliked self-help treatments that involved taking medication and were particularly concerned about painkillers in combination. Steam inhalation was viewed as only moderately helpful for mild symptoms.

Conclusion: Delayed prescribing is acceptable no matter how the delay is operationalized, but explanation of the rationale is needed and care taken to minimise mixed messages about the severity of illnesses and causation by viruses or bacteria. Better access is needed to good natural history information, and the signs and symptoms requiring or not requiring GP advice. Significant concerns about paracetamol, ibuprofen and steal inhalation are likely to need careful exploration in the consultation.

Article summary

Strengths and limitations of this study (related to methods)

1. A range of views were solicited and data saturation was reached.
2. Where there is existing literature this study resonates with it,
3. The interviews permitted open exploration of delayed prescribing which is novel
4. Interviews are reports and not a window onto the actual events/experiences reported

For peer review only - http://bmjopen.bmj.com/site/about/guidelines.xhtml

5. Trial participation may have led to sample of participants particularly interested in this research and may not represent views of ‘typical’ patients.

Background

The overuse of antibiotics can contribute to the spread of resistant bacteria[1-2]. This problem is currently on the increase and has been identified by the World Health Organisation as a serious issue which must be addressed with urgency[3]. Despite evidence to suggest the limited benefit of antibiotics in the treatment of respiratory tract infections (RTI) in particular[4-5], RTIs account for >60% of all antibiotic prescriptions in primary care[6] and primary care accounts for 80% of human antibiotic consumption[7]. The delayed prescribing of antibiotics is a technique which may help to reduce unnecessary prescribing in primary care[8,9]. The method is recommended by the NICE guidelines[6] and involves a prescription being issued by the GP for patient use at a later date if symptoms do not improve, or if they worsen. This can be delivered using a number of techniques which include four common methods – providing an antibiotic prescription to the patient, dated on the day of consultation, providing a post-dated prescription, the patient telephoning the practice if they meet issuing criteria for the practice to then issue a prescription, and the prescription being left at the surgery reception for the patient to collect if the patient feels it necessary. In addition, the use of self-help over-the-counter medications and techniques can also be used to help relieve symptoms of RTI without the use of antibiotics: these include the use of paracetamol, ibuprofen and steam inhalation[10-12].

The PIPS (Pragmatic Ibuprofen Paracetamol and Steam) trial aimed to assess the effectiveness of four common methods of delayed prescribing outlined above, and the use of analgesics (paracetamol; ibuprofen or both) and steam inhalation for RTI, using a randomised factorial trial design[13]. This paper reports on a qualitative study nested within the PIPS trial in order to determine the feasibility and acceptability of these management strategies from the perspective of participating patients and to investigate factors that are currently influencing their decisions to consult a GP.

Methods

Participants and procedure

For peer review only - http://bmjopen.bmj.com/site/about/guidelines.xhtml

Adult participants were recruited from a sample of patients presenting with RTI participating in the PIPS trial who had consented to being contacted for an interview. The participants were recruited from areas across Hampshire. Interview participants were purposively sampled to include a maximum possible variety of trial stage (from beginning to end of trial), trial arm (e.g. delayed prescribing method), age and gender. Participants were recruited by telephone and written consent was obtained.

Interviews

Trained female interviewers (LM, AN, JW) conducted face to face (n=10) and telephone interviews (in order to cover a wide geographic area, n=10), with each lasting approximately half an hour. All interviews took place at the GP clinic or the participant's home, and were audio–recorded and transcribed verbatim. Our epistemological position is best characterised by subtle realism[14]. Qualitative interviews provided the best method for gathering insights into participants' individual views about and experiences of treatments for RTI. A semi-structured interview guide (Appendix 1) included key topic areas while also providing flexibility to explore unanticipated issues. AN and JW were medical students at the time of data collection and supervised by senior researchers (GL and PL). Everyone who consented to participate was interviewed.

Analysis

Inductive thematic analysis[15] was conducted on all transcripts to determine factors which influence patients' decision to consult a GP or use an alternative treatment for RTI, as well as being open to themes outside of the core aims of the study. Following immersion in the transcripts, familiarisation was achieved and patterns and prominent themes which consistently occurred in the data were identified and labelled with codes. Each code label referred directly to the operationalisation of the theme content. A label and full descriptive definition was then provided for each theme. Codes and definitions were refined during a continuing process, which involved themes being linked, grouped, moved, re-labelled, added and removed to produce a set of themes and subthemes and a coding manual, which adequately fitted and thoroughly explained the data. The coding was iteratively developed across authors (led by LM and GL) and adjustments made where appropriate based on discussion. The analysis process showed that saturation had been reached as no new information was emerging from the later transcripts.

For peer review only - http://bmjopen.bmj.com/site/about/guidelines.xhtml

Findings

Participants:

Twenty people participated, with ages ranging from 18-74 (mean age 51), and 70% (N=14) were female. In addition approximately 50% (N=11) of participants had completed the trial at the time of interview. All patients had experienced at least two or three different treatment options for RTI either as part of the trial or in the past.

Themes:

Thematic analysis identified a total of five key themes relating to patients' views of different management approaches, and factors which may influence a patient's decision to use a self-help treatment (paracetamol/ibuprofen/steam inhalation) or consult with a GP for the treatment of a RTI.

Table 1: Themes identified in analysis

Themes	Sub-themes	
**1. Perceptions of illness**		-Duration of symptoms<br>-Perceived signs of severity<br>-Quality of life impact
**2. Advice from others**		-Acceptance of GP advice<br>-Consideration of alternative advice
**3. Perceptions of antibiotics**		-Antibiotics have unpleasant side-effects<br>-Concerns and misunderstandings about resistance<br>-Antibiotics needed for specific cases
**4. Perceptions of self-help treatment**		-Treatment duration short and irregular<br>-Experience of effective treatment
	**Paracetamol and ibuprofen**	-Apprehension of combination medication<br>-General dislike of medication

For peer review only

		-Concerns of medication side-effects<br>-Medication acceptable for pain relief
	**Steam inhalation as a limited technique**	-Only beneficial if not severe<br>-Provides short term relief<br>-Relieves but not cures
**5. Perceptions of delayed prescribing**		-Viewed as a positive option<br>-Acceptance of GP recommended method<br>-Confusion of delayed prescribing role.

In the following sections we summarise themes 1 and 2 for context before reporting in depth on themes 3-5, describing each in turn, using exemplary quotations for illustrative purposes (participant number is shown in parentheses). Findings are discussed in relation to the most prevalent and influential themes outlined by participants.

1 and 2: Perceptions of illness severity, and advice from others

Participants' views on the severity of their condition related to the evaluation of the duration of symptoms. Participants signalled that they were mostly unaware of the natural history, and specific symptoms (such as breathing difficulty) were often highlighted as indicators that their condition might be severe, resulting in the decision to consult.

> *"I think it was because it was - I could feel or could hear a creaking or croaking in the chest cavity…So that said to me, right we need to go and get something to deal with it."(PL7)*

Participants reported making decisions on how to treat their condition based on advice from both GPs and alternative professional and lay sources which included pharmacists, family members and the media.

> *"I do the Karvol inhalation, my patent remedy…. That's an old one from my mother, so it's a long, long time ago, but….I think that'd be the best treatment"(P03)*

For peer review only

3. Perceptions of antibiotics

A patient's decision on how to treat their condition was reported as being influenced by their perceptions of antibiotics, such as relating to beliefs about unpleasant side effects and concerns about resistance. Patients who described unpleasant side-effects associated with the use of antibiotics tended to report reluctance to take them, unless absolutely necessary.

> *"I don't like taking antibiotics because, after all, whatever it does to the system in relation to the bowel side of it, it destroys all the bacteria there anyhow so it's better to, if you can get away without taking them, the better"(PL08)*

In line with existing literature that identified people may believe the body to be resistant rather than the bacteria[16], many participants reported concerns about antibiotic resistance, which influenced their decision on how to best treat their condition and symptoms. Participants reported the belief that antibiotics were necessary in some specific situations and circumstances, such as patients with co-morbidities.

> *"Well in my own particular case … I almost feel that if I do go the doctor's with a nasty cough or some sort of … you know, nasal blockage or ear ache, I almost thought I ought to be given antibiotics as a matter of course because of my asthma" (PL7)*

4. Perceptions of self-help treatment

Participants' perceptions surrounding the potential benefits and limitations of self-help treatments for RTI influenced their decision to try these or visit their GP. They reported that in general self-help treatments were used for short periods of time at irregular intervals, in contrast with the likely prescribed management approach offered by a GP to take pain relief at regular intervals.

> *"My GP did say that managed pain is better, so take it regularly, but I tend not to do that; perhaps have some in the morning and if I feel really ropey in the afternoon, but then definitely in the evening." (PL8)*

For peer review only

In general, participants reported a dislike for self-help treatments which involved taking medication. However, it is interesting to note that despite views of disliking these medications, participants were more likely to use them if the GP advised it, and would be less likely to try these without such advice. Interviewees reported concerns and worries relating to possible side effects which taking paracetamol or ibuprofen may cause.

> *"Yes, isn't there a problem with that [ibuprofen] in relation to blood? Yes, there's something I've heard about ibuprofen, that particular medication, which I didn't think was too good...I would not want to take it in that sense."(PL01)*

Participants reported being apprehensive about taking a combination of ibuprofen and paracetamol together. The combination of medication was construed by some as somehow 'risky' when compared to taking a single type of medication.

> *"Well I should have thought that was a bit of a lethal cocktail together but I don't know; if it was up to me I wouldn't want to do that."(PL08)*

Despite concerns relating to taking paracetamol or ibuprofen for the treatment of RTI, participants reported that they were happy to take these for the use of pain relief. This was a familiar usage and appeared to be considered as more acceptable than using them for an RTI. Most reported steam inhalation could relieve symptoms to a degree, however it was perceived as only providing short-term relief from symptoms and that it would not assist in curing the underlying condition. Therefore if symptoms were considered as serious, using steam inhalation was not viewed as a potential overall treatment option.

> *"I don't think that they actually cure me, but I think they give me a little bit of relief from congestion." (PL09)*

5. Perceptions of delayed prescribing

Delayed prescribing was generally viewed as a positive technique and patients reported being happy to accept whichever method of delayed prescribing the GP recommended. None of the participants expressed disappointment at not receiving an antibiotic prescription for immediate use. However, there was some confusion reported as to the role of delayed

For peer review only - http://bmjopen.bmj.com/site/about/guidelines.xhtml

prescribing. It was generally viewed by participants as a positive technique which could give them reassurance of having access to a prescription just in case. The main benefit was seen as the patient having some decision in their own treatment while preventing them from unnecessarily taking antibiotics.

> *"I don't mind delayed prescribing. I mean, I would rather have them and know that I've got them and, yes, if I don't need them, then I wouldn't take them, but I like the safety [of a delayed prescription], knowing that I've got them, because more often than not, it doesn't go away" (P05)*

Although happy to accept delayed prescribing as a treatment option, many participants were confused as to the purpose of the technique. Some reported confusion because they had been told their condition was viral yet had still been issued a delayed prescription, indicating the rationale for delayed prescribing may need to be clearer.

> *"…either you have or haven't got an infection that either does or doesn't need treating. And if it needs treating it needs treating now, if not it doesn't" (PA7)*

Some expressed the view that the technique might be driven by economic reasons.

> *"I am more and more wondering if medication is being withheld because of expenses…surgeries have to make a living, they have to work within a budget…" (PA3)*

Discussion

Main findings

Overall, the findings suggest participants were aware of the limitations of antibiotics and do not wish to receive them every time they consult a GP for RTIs. There was some confusion about the rationale for delayed prescribing with a particular risk of mixed messages, whereby on the one hand some expressed the conflict between being told their condition did not warrant immediate treatment because it may not be a bacterial infection, whilst on the other hand it might at some point require antibiotic treatment. This is a complicated message for

For peer review only

GPs to deliver and for patients to receive, interpret and understand. It may be that GPs should not discuss viruses during the consultation, and rather emphasise that most types of RTIs settle on their own without the need to antibiotics. Some participants suspected that delayed prescribing was primarily driven by economic decisions and the need to cut spending, but most participants viewed delayed prescriptions positively, suggesting they were happy to accept their GP's recommendation. That said, what cannot be emphasised enough is the clear need for GPs and their patients to negotiate the delayed disposal and to render transparent the rationale for a delay.

Participants expressed concerns over self-help that involved taking medication, particularly painkillers in combination for the treatment of RTI. When patients did take medication some indicated irregular use of them, which contrasts with a need for regular and continued dosing, sometimes when symptoms appear to have reduced. Just taking 'drugs' when they feel they need to is intuitively logical and may signal a need for clearer information that people could get better and feel better more quickly if they take medication as recommended. Indeed, it seemed overall, despite reservations, participants conveyed an overall stance of a willingness to accept advice to use painkillers if recommended by their GP. Some interviewees seemed to require authorisation from their GP to try over-the-counter remedies: this may link to a misconception that analgesics do not help for respiratory symptoms. Whilst steam inhalation was viewed as an acceptable treatment participants generally described this method as providing short-term relief from non-severe symptoms. The decision to consult a GP was initially influenced by patient perceptions of the natural history, a variety of personal indicators, and beliefs largely unrelated to documented natural history indices of severity.

Comparison with existing literature

Participants reported being aware of the limitations of antibiotics and not expecting to receive them every time they went to the GP with a RTI which supports previous research, and often contrasts with GP perceptions of high levels of patient pressure to prescribe[8]. However, the self-selected nature of the sample interviewed is likely to mean they were more open to exploring alternatives to antibiotics[17]. There is still considerable room for patients to have a better understanding of the limited benefit from antibiotics, such a patient information leaflets, given the evidence that beliefs in the effectiveness of antibiotics are still strong[13,18]. There is also room to ensure potential misunderstandings are managed well when a no-

For peer review only

prescribing disposal is selected. For example, whilst not common, some interview accounts suggested that not prescribing antibiotics can be understood (at least in part) to be a resource saving strategy rather than a clinically driven one. Tonkin-Crine et al.[19] also found that older adults may hold such a view. In addition, the perception of a delayed script as potentially contradictory to GP advice that an antibiotic prescription may not be required also needs to be tackled when a prescribing decision is negotiated. Patients reported high levels of concordance with GP advice across all treatment options, including all methods of delayed prescribing. Previous studies suggest high patient compliance/concordance with GP advice is strongly associated with doctor-patient agreement, which is facilitated by patient understanding of treatment benefits[20]. Data presented here suggested participants would have been happy to accept a delayed prescription: these perceptions were supported by the quantitative satisfaction data from the trial[13]. Previous research identified patient satisfaction with delayed prescribing[21], but also a subgroup of patients who had expected antibiotics and were disappointed when they did not receive a prescription for immediate use. The current study in contrast did not find a group of patients who were disappointed at not receiving antibiotics. Speculatively, this may, in part, be associated with societal changes and the extensive publicity about antibiotic resistance. However, this also may reflect selection bias since trial patients may represent a sub-group who are interested in research, have more awareness of issues surrounding antibiotic prescribing, and are more open to trying different approaches and therefore happier to accept new advice or techniques. Furthermore, since all participants were taking part in a trial it is likely that GPs provided a detailed explanation of their treatment decision, using structured advice sheets, which is unlikely to happen in routine practice [22].

Implications for clinical practice

Overall, participants reported delayed prescribing to be an acceptable treatment option. Previous research[23] has suggested that patient understanding of the rationale for a particular treatment can increase patient satisfaction. In this study delayed prescribing was delivered using structured advice sheets detailing the expected natural history; benefits/disbenefits of antibiotics; how long to delay; and safety netting/symptom advice. The findings suggest good concordance and high satisfaction should accompany a consistent and thorough explanation of the approach. Participants did not display a strong preference of any particular method of

For peer review only

delayed prescribing, which suggests any approach is likely to be acceptable in practice, as long as a clear explanation is provided. .

Findings suggest participants were concerned about using painkillers as a treatment method for RTI. They reported concerns relating to potential hazards of combining analgesics, which links with existing evidence of low analgesic concordance rates in patients with RTI[24]. However, interviewees did report that they thought paracetamol and ibuprofen were, at least in isolation, acceptable treatments for pain relief. The results of the main trial suggest that advice to consume regular ibuprofen either alone or in combination may be associated with worse outcomes and therefore a preference for paracetamol would be the recommended approach in future[13]. This finding suggests that some analgesics can be viewed as feasible treatment options for patients, but education and explanation of the justification and possible benefits/dis-benefits for these medications need to be shared with patients, as well as emphasising the role of pharmacists in terms of offering information and support.

This study shows how patients' decisions to consult a GP were often related to perceived indicators of illness severity which were more often incorrect than correct, as well as beliefs relating to whether they thought they had caught the same condition as other people they knew. These findings are consistent with Leventhal's (1984) model of illness representations[25] which suggests that patients' beliefs relating to the cause, identity, duration and controllability of an illness are all likely to influence the way in which they respond to the condition (e.g. consulting a GP). The GRACE INTRO European study used this model to develop a patient booklet and findings indicated it was well received by patients and gave them new information about illness duration and how to self care[19,26]. Other resources have also been developed to improve patient understanding of RTIs in primary care[27,28,29]. As most of the indicators that patients used to determine illness severity may not warrant a GP consultation, it suggests a need to review the clarity of patient information (e.g. providing clear examples of symptoms and illness duration that would ordinarily merit a visit to the GP, and those which are not a cause for worry). The promotion of this information within the community, as well as dealing with the common misconception that antibiotic medication may help coughs and colds[17] could help to reduce unnecessary GP consultations in the future.

For peer review only

Strengths and limitations

One of the key strengths of the study is that all patients interviewed had experienced at least two or three different treatment options for RTI either as part of the trial or in the past. This included antibiotics, delayed prescribing, paracetamol, ibuprofen, and steam inhalation. Therefore, the interviews provided opportunity for discussion of the various treatment options with a targeted information-rich sample. This strengthens the findings and allows stronger conclusions to be drawn.

However, the sample of participants who took part in the interviews may have limited the scope of the findings somewhat, as they had all participated in the PIPS trial. This may have led to a sample of participants who were particularly interested in research of this nature and may not have represented views held by 'typical' patients, particularly views on antibiotic resistance. Also, the participants had all consulted for an RTI and therefore may have different views than individuals who may not consult for such symptoms. Whilst having incomplete information on patient characteristics including information on people who declined participation is a limitation of this study, the research does provides novel and relevant findings.

Conclusion

The findings suggest that delayed prescribing appears to be an acceptable technique, as long as the method of delayed prescribing is described well to optimise patient understanding of the rationale. Increased patient education relating to the safety and justification of using paracetamol for the treatment of RTI is likely to increase the acceptability of analgesics. In addition, enhanced approaches to sharing information with patients- relating to the natural history of illness, the appropriate signs and symptoms requiring GP advice, and those symptoms and signs which do not require urgent attention – could help reduce unnecessary consultations.

Acknowledgements

We are grateful to all the general practitioners and practice nurses and particularly the patients who gave their time to this study.

PIPS Investigators

University of Southampton: Paul Little, Ian Williamson, Mike Moore, Mark Mullee, Jo Kelly, Julie Hooper, Lisa McDermott, Geraldine Leydon, Ben Holdstock-Brown*, Amanda Nagle*, Jennifer White* (*medical students at the time); Samantha Hall, Patient and Public (PPI) representative.

Author contribution

GM Leydon led the qualitative work as part of the PIPs trial. P Little secured research funding/and acted as overall PI of the PIPs trial. J Kelly facilitated recruitment. L McDermott, A Nagle, and J White collected interview data. All authors were involved in/commented on data analysis (led by GM Leydon and L McDermott). A Halls and GM Leydon developed the manuscript, to which all authors contributed.

Funding statement

This article presents independent research funded by the National Institute for Health Research (NIHR) under its Programme Grants for Applied Research programme (grant ref No RP-PG-0407-10098). The views expressed are those of the author(s) and not necessarily those of the NHS, the NIHR or the department of Health.

Competing interests statement

There are no competing interests to report.

Ethical approval

The study was approved by Southampton and Southwest MREC number 06/Q1702/154 and all participants gave informed consent.

Data sharing

No additional data available.

References

1. Dagan R, Leibovitz E, Greenberg D, Yagupsky P, Fliss DM, Leiberman A. Dynamics of pneumococcal nasopharyngeal colonization during the first days of antibiotic

For peer review only

treatment in pediatric patients. *J Ped Infect Dis* 1998; 17:880-5.

2. Costelloe C, Metcalfe C, Lovering A, Mant D, Hay A. Effect of antibiotic prescribing in primary care on antimicrobial resistance in individual patients: Systematic review and meta-analysis. *BMJ* 2010;340:c.2096.
3. World Health Organisation. The evolving threat of antimicrobial resistance: Options for action. WHO; 2012.
4. Little P, Watson L, Morgan S, Williamson I. Antibiotic prescribing and admissions with major suppurative complications of respiratory tract infections: a data linkage study. *Br J Gen Pract* 2002;52(476):187-90.
5. Macfarlane J, Holmes W, Gard P, Thornhill D, Macfarlane R, Hubbard R. Reducing antibiotic use for acute bronchitis in primary care: blinded, randomised controlled trial of patient information leaflet. *BMJ* 2002; 324:91-4.
6. NICE Guideline Development Group. Respiratory tract infections – antibiotic prescribing: Prescribing of antibiotics for self-limiting respiratory tract infections in adults and children in primary care. London: NICE; 2008
7. ESPAUR report Public Health England. English surveillance programme for antimicrobial utilisation and resistance (ESPAUR) 2010-2014. London: Public Health England; 2015.
8. Little P, Rumsby K. Kelly J, Watson L, Moore M, Warner G, et al. Information leaflet and antibiotic prescribing strategies for acute lower respiratory tract infection: a randomised controlled trial. *JAMA* 2005;293:3029-35.
9. De la Poza Abad MD et al: Prescription strategies in acute uncomplicated respiratory infections: A randomized clinical trial. *JAMA Intern Med.* 2016;176(1):21-29. doi:10.1001/jamainternmed.2015.7088
10. Hollinghurst S, Redmond N, Costelloe C, Montgomery A, Fletcher M, Peters TJ, Hay AD. Paracetamol plus ibuprofen for the treatment of fever in children (PITCH). *BMJ* 2008;337:a1490.
11. Singh M, Heated, humidified air for the common cold. *Cochrane Library* 2004;2:CD001728.
12. Burke P, Bain J, Robinson, D, Dunleavey J. Acute red ear in children: controlled trial of non-antibiotic treatment in general practice. *BMJ* 1991;303:558-562
13. Little P, Moore M, Kelly J et al. Ibuprofen, paracetamol, and steam for patients with respiratory tract infections in primary care: pragmatic randomised factorial trial *BMJ* 2013;347:f6041.

For peer review only

14. Hammersley M: *What's Wrong With Ethnography?* London: Routledge, 1992.
15. Braun V, and Clarke V, Using thematic analysis in psychology. *Qual Res in Psych* 2006; 3:77-101.
16. Brookes-Howell L, Elwyn G, Hood K, Wood F, Cooper L, Goossens H, Ieven M, Butler CC. 'The body gets used to them': patients' interpretations of antibiotic resistance and the implications for containment strategies *J Gen Intern Med* 2012;27(7):766-72.
17. McNulty CAM, Nichols T, French DP, Joshi P, Butler CC. 'Expectations for consultations and antibiotics for respiratory tract infection in primary care: the RTI clinical iceberg *Brit J Gen Pract* 2013;63(612):e.429-e.436.
18. McNulty CAM, Boyle P, Nichols T, Clappison P, and Davey P. The public's attitudes to and compliance with antibiotics. *J Antimicrob Chemo* 2007; 60 (suppl 1):i63-i68.
19. Tonkin-Crine S, Anthierens S, et al. 'Exploring patients' views of primary care consultations with contrasting interventions for acute cough: a six-country European qualitative study, npj *Primary Care Respiratory Medicine* 2014;24:14026.
20. Kerse N, Buetow S, Mainous AG, Young, G, Coster G, Arroll B. 'Physician-patient relationship and medication compliance: A primary care investigation. *Ann Fam Med* 2004;2:455-461.
21. Arroll BA, Goodyear-Smith F, Thomas DR, Kerse N. Delayed antibiotic prescriptions: what are the experiences and attitudes of physicians and patients? *J Fam Pract* 2002;51;954-9.
22. Little P, Moore M, Kelly J, Williamson I, Leydon G, McDermott L, Mullee M, Stuart B. Delayed antibiotic prescribing strategies for respiratory tract infections in primary care: pragmatic, factorial, randomised controlled trial. *BMJ* 2014; 14:348
23. Francis NA, Phillips R, Wood F, Hood K, Simpson S, Butler CC. Parents' and clinicians' views of an interactive booklet about respiratory tract infections in children: a qualitative process evaluation of the EQUIP randomised controlled trial. *BMC Family Practice* 2013;14:182.
24. Little P, Gould C, Williamson I, Moore M, Warner G, Dunleavey J. A pragmatic randomised controlled trial of two prescribing strategies for acute otitis media. BMJ 2001;332:336-42.
25. Leventhal H, Nerenz DR, Steel DJ. Illness representations and coping with health threats. In: Baum A, Taylor SE, Singer JK, editors. Handbook of Psychology and Health: Social psychological aspects of health. Hillsdale, NJ: Earlbaum, 1984.

For peer review only

26. Yardley L, Douglas E, Athierens S, Tonkin-Crine S, O'Reilly G, Stuart B. et al. Evaluation of a web-based intervention to reduce antibiotic prescribing for LRTI in six European countries: quantitative process analysis of the GRACE/INTRO randomised controlled trial *Implementation Science* 2013;8:134

27. Francis N, Butler C, Hood K, Simpson S, Wood F, Nuttall J. Effect of using an interactive booklet about childhood respiratory tract infections in primary care consultations on reconsulting and antibiotic prescribing: a cluster randomised controlled trial *BMJ* 2009;339:b2885

28. TARGET Treating Your Infection patient leaflet version 5, available at http://www.rcgp.org.uk/clinical-and-research/toolkits/target-antibiotics-toolkit/patient-information-leaflets.aspx.

29. Cabral C, Ingram J, Lucas P. TARGET Children with Cough leaflet, available at http://child-cough.bristol.ac.uk.

For peer review only

Appendix 1

Introduction: Topics to be covered

The aim of this interview is to understand (1) the diagnosis and management of respiratory tract infections (RTIs) and your understanding of what has caused your infection (2) Patients' views on using paracetamol, ibuprofen and antibiotics.

If you could start at the beginning and tell me what led you/your child coming to see the doctor/nurse with your/your child's infection?

Prompts: Severity? Duration? Self-help- tried, if so what?

Previous experience of sore throat?
Previous experience of seeing doctor and getting treatment?
Family/friend/social support network?

We are interested in patient views on what causes RTI. Could you tell me what you think may have caused your illness or where you got it from?

Prompt: Bacteria? (If so, where from?) Virus? Weak system? Past/family history?

Could you please tell me if you have previously used paracetamol?

Frequency? Reason?

What are your views about taking paracetamol?

Prompt: Is taking full doses a problem or harmful in any way- e.g. is fever 'good'? Concerns?

Have you ever heard about problems with paracetamol?

e.g. poison; side effects

Do you remember the advice about paracetamol?

Prompt: Clear advice? Did you use it regularly rather than on demand? Did you use full doses?

If you stopped using it, why? (e.g. severity of symptoms settling, otherwise coping?)

Have you previously used ibuprofen? How do you feel about it?

Prompt: Is taking full doses a problem or harmful in any way- e.g. is fever 'good'?
Have you heard about problems with ibuprofen? (e.g. ulcers; side effects?)

For peer review only

Do you remember the advice about ibuprofen?
Did you use it regularly rather than on demand? Did you use full doses?
If you stopped using it, why? (e.g. severity of symptoms settling, otherwise coping?)

Have you previously had antibiotics?
How did you feel about them?
Have you heard about problems with antibiotics (e.g. resistance; side effects?)

Do you remember the advice about antibiotics?
Were you advised to wait, if so, how long?
What was your experience of waiting?

Have you come across delayed prescribing before?
Prompt: Provide more information if participant unsure.

Outline the four common methods of delayed prescribing.

How did you feel about the method of delayed prescribing you were offered?
OR
How do you think you would feel about being offered delayed prescribing if you/your child consulted again?

If you started antibiotics, why? (e.g. severity of symptoms, general deterioration in well-being).

In your consultation with the doctor, how did you feel the doctor responded to your infection?"

Prompt: How did you feel about the consultation you had? Sympathetic? Enough information? Clear advice?

Were you given other self-help advice? If so, what did you think about it?

What other self-help measures did you use (e.g. tepid sponging?)

Conclusion

Are there any relevant issues we haven't covered that you would like to mention?

Are there any questions you would like to ask me?

For peer review only

COREQ (COnsolidated criteria for REporting Qualitative research) Checklist

A checklist of items that should be included in reports of qualitative research. You must report the page number in your manuscript where you consider each of the items listed in this checklist. If you have not included this information, either revise your manuscript accordingly before submitting or note N/A.

Topic	Item No.	Guide Questions/Description	Reported on Page No.
**Domain 1: Research team and reflexivity**			
Personal characteristics			
Interviewer/facilitator	1	Which author/s conducted the interview or focus group?	4
Credentials	2	What were the researcher's credentials? E.g. PhD, MD	4
Occupation	3	What was their occupation at the time of the study?	4
Gender	4	Was the researcher male or female?	4
Experience and training	5	What experience or training did the researcher have?	4
Relationship with participants			
Relationship established	6	Was a relationship established prior to study commencement?	N/A
Participant knowledge of the interviewer	7	What did the participants know about the researcher? e.g. personal goals, reasons for doing the research	N/A
Interviewer characteristics	8	What characteristics were reported about the inter viewer/facilitator? e.g. Bias, assumptions, reasons and interests in the research topic	N/A
**Domain 2: Study design**			
Theoretical framework			
Methodological orientation and Theory	9	What methodological orientation was stated to underpin the study? e.g. grounded theory, discourse analysis, ethnography, phenomenology, content analysis	4
Participant selection			
Sampling	10	How were participants selected? e.g. purposive, convenience, consecutive, snowball	3
Method of approach	11	How were participants approached? e.g. face-to-face, telephone, mail, email	3
Sample size	12	How many participants were in the study?	4
Non-participation	13	How many people refused to participate or dropped out? Reasons?	Unknown
Setting			
Setting of data collection	14	Where was the data collected? e.g. home, clinic, workplace	4
Presence of non-participants	15	Was anyone else present besides the participants and researchers?	No
Description of sample	16	What are the important characteristics of the sample? e.g. demographic data, date	4
Data collection			
Interview guide	17	Were questions, prompts, guides provided by the authors? Was it pilot tested?	4 and appendi
Repeat interviews	18	Were repeat inter views carried out? If yes, how many?	No
Audio/visual recording	19	Did the research use audio or visual recording to collect the data?	4
Field notes	20	Were field notes made during and/or after the inter view or focus group?	
Duration	21	What was the duration of the inter views or focus group?	3
Data saturation	22	Was data saturation discussed?	4
Transcripts returned	23	Were transcripts returned to participants for comment and/or	No

For peer review only

Topic	Item No.	Guide Questions/Description	Reported on Page No.
		correction?	
**Domain 3: analysis and findings**			
Data analysis			
Number of data coders	24	How many data coders coded the data?	4
Description of the coding tree	25	Did authors provide a description of the coding tree?	No
Derivation of themes	26	Were themes identified in advance or derived from the data?	4
Software	27	What software, if applicable, was used to manage the data?	N/A
Participant checking	28	Did participants provide feedback on the findings?	N/A
Reporting			
Quotations presented	29	Were participant quotations presented to illustrate the themes/findings? Was each quotation identified? e.g. participant number	5 onwards
Data and findings consistent	30	Was there consistency between the data presented and the findings?	5
Clarity of major themes	31	Were major themes clearly presented in the findings?	5
Clarity of minor themes	32	Is there a description of diverse cases or discussion of minor themes?	5

Developed from: Tong A, Sainsbury P, Craig J. Consolidated criteria for reporting qualitative research (COREQ): a 32-item checklist for interviews and focus groups. *International Journal for Quality in Health Care*. 2007. Volume 19, Number 6: pp. 349 – 357

Once you have completed this checklist, please save a copy and upload it as part of your submission. DO NOT include this checklist as part of the main manuscript document. It must be uploaded as a separate file.

For peer review only

A qualitative interview study of antibiotics and self-management strategies for respiratory infections in primary care.

Journal:	*BMJ Open*
Manuscript ID	bmjopen-2017-016903.R2
Article Type:	Research
Date Submitted by the Author:	10-Jul-2017
Complete List of Authors:	McDermott, Lisa; King's College London, Primary Care and Public Health Sciences<br>Leydon, Gerry; University of Southampton, Primary Care and Population Sciences<br>Halls, Amy; University of Southampton, Primary Care and Population Sciences<br>Kelly, Jo; University of Southampton, Primary Care and Population Sciences<br>Nagle, Amanda; University of Southampton, Primary Care and Population Sciences<br>White, Jennifer; University of Southampton, Primary Care and Population Sciences<br>Little, Paul; University of Southampton, Primary Care and Population Science;
**Primary Subject Heading**:	General practice / Family practice
Secondary Subject Heading:	Qualitative research
Keywords:	antibiotics, prescribing, respiratory tract infection, self-help

SCHOLARONE™ Manuscripts

A qualitative interview study of antibiotics and self-management strategies for respiratory infections in primary care.

Authors:

McDermott L*±, Leydon GM**±, Halls A**, Kelly J**, Nagle A**, White J**, Little P**, on behalf of the PIPS investigators.

*Primary Care and Public Health Sciences, King's College, London
614, 6th Floor, Capital House, 42 Weston Street, London, SE1 3QD

**Primary Care and Population Sciences, Faculty of Medicine, University of Southampton
Aldermoor Health Centre, Aldermoor Close, Southampton, SO16 5ST

±These authors are to be regarded as Joint First Authors.

Corresponding author: GM Leydon gerry@soton.ac.uk

Key words: Antibiotics, prescribing, Respiratory Tract Infection, self-help

For peer review only

Abstract

Objective: To explore perceptions of illness, the decisions to consult, and the acceptability of delayed antibiotic prescriptions and self-help treatments for respiratory tract infections (RTIs).

Design: Qualitative semi-structured interview study.

Setting: UK Primary care.

Participants: 20 adult patients who had been participating in the 'PIPS' (Pragmatic Ibuprofen Paracetamol and Steam) trial in the South of England.

Method: Semi-structured, telephone interviews were conducted with participants to explore their experiences and views on various treatments for RTI.

Results: Participants had concerns about symptoms which were not clinically serious and were mostly unaware of the natural history of RTIs, but were aware of the limitations of antibiotics and did not expect them with every consultation. Most viewed delayed prescriptions positively and had no strong preference over which technique is used to deliver the delayed antibiotic, but some patients received mixed messages, such as being told their infection was viral then being given an antibiotic, or were sceptical about the rationale. Participants disliked self-help treatments that involved taking medication and were particularly concerned about painkillers in combination. Steam inhalation was viewed as only moderately helpful for mild symptoms.

Conclusion: Delayed prescribing is acceptable no matter how the delay is operationalized, but explanation of the rationale is needed and care taken to minimise mixed messages about the severity of illnesses and causation by viruses or bacteria. Better access is needed to good natural history information, and the signs and symptoms requiring or not requiring GP advice. Significant concerns about paracetamol, ibuprofen and steal inhalation are likely to need careful exploration in the consultation.

Article summary

Strengths and limitations of this study (related to methods)

1. A range of views were solicited and data saturation was reached.
2. Where there is existing literature this study resonates with it,
3. The interviews permitted open exploration of delayed prescribing which is novel
4. Interviews are reports and not a window onto the actual events/experiences reported

For peer review only - http://bmjopen.bmj.com/site/about/guidelines.xhtml

5. Trial participation may have led to sample of participants particularly interested in this research and may not represent views of 'typical' patients.

Background

The overuse of antibiotics can contribute to the spread of resistant bacteria[1-2]. This problem is currently on the increase and has been identified by the World Health Organisation as a serious issue which must be addressed with urgency[3]. Despite evidence to suggest the limited benefit of antibiotics in the treatment of respiratory tract infections (RTI) in particular[4-5], RTIs account for >60% of all antibiotic prescriptions in primary care[6] and primary care accounts for 80% of human antibiotic consumption[7]. The delayed prescribing of antibiotics is a technique which may help to reduce unnecessary prescribing in primary care[8,9]. The method is recommended by the NICE guidelines[6] and involves a prescription being issued by the GP for patient use at a later date if symptoms do not improve, or if they worsen. This can be delivered using a number of techniques which include four common methods – providing an antibiotic prescription to the patient, dated on the day of consultation, providing a post-dated prescription, the patient telephoning the practice if they meet issuing criteria for the practice to then issue a prescription, and the prescription being left at the surgery reception for the patient to collect if the patient feels it necessary. In addition, the use of self-help over-the-counter medications and techniques can also be used to help relieve symptoms of RTI without the use of antibiotics: these include the use of paracetamol, ibuprofen and steam inhalation[10-12].

The PIPS (Pragmatic Ibuprofen Paracetamol and Steam) trial aimed to assess the effectiveness of four common methods of delayed prescribing outlined above, and the use of analgesics (paracetamol; ibuprofen or both) and steam inhalation for RTI, using a randomised factorial trial design[13]. This paper reports on a qualitative study nested within the PIPS trial in order to determine the feasibility and acceptability of these management strategies from the perspective of participating patients and to investigate factors that are currently influencing their decisions to consult a GP.

Methods

Participants and procedure

For peer review only

Eligible adults who had consented to participating in the main PIPS trial, and to being contacted by a researcher to discuss participating in an interview, were consecutively sampled, with on-going monitoring for variation and prioritising the selection of individuals who would enhance the diversity of our sample where possible. This included a maximum possible variety of trial stage (from beginning to end of trial), trial arm (e.g. delayed prescribing method), age and gender, to ensure we captured any potential variation in views. Participants had been recruited to the main trial through primary care by either GPs or nurses, and were randomised to one of twelve basic groups. Participants were recruited for this interview study by telephone from areas across Hampshire, a minimum of two weeks after recruitment and written consent was obtained.

Interviews

Trained female interviewers (LM, AN, JW) conducted face to face (n=10) and telephone interviews (in order to cover a wide geographic area, n=10), with each lasting approximately half an hour. All interviews took place at the GP clinic or the participant's home, and were audio–recorded and transcribed verbatim. Our epistemological position is best characterised by subtle realism[14]. Qualitative interviews provided the best method for gathering insights into participants' individual views about and experiences of treatments for RTI. A semi-structured interview guide (Appendix 1) included key topic areas while also providing flexibility to explore unanticipated issues. Participants were asked about their experiences of RTIs to date, not solely within the context of the trial. They were also asked about different management strategies, their understanding of antibiotics, and factors currently influencing their decision to consult a GP. AN and JW were medical students at the time of data collection and supervised by senior researchers (GL and PL). Everyone who consented to participate was interviewed.

Analysis

Inductive thematic analysis[15] was conducted by hand on all transcripts to determine factors which influence patients' decision to consult a GP or use an alternative treatment for RTI, as well as being open to themes outside of the core aims of the study. Following immersion in the transcripts, familiarisation was achieved and patterns and prominent themes which consistently occurred in the data were identified and labelled with codes. Each code label

For peer review only

referred directly to the operationalisation of the theme content. A label and full descriptive definition was then provided for each theme. Codes and definitions were refined during a continuing process, which involved themes being linked, grouped, moved, re-labelled, added and removed to produce a set of themes and subthemes and a coding manual, which adequately fitted and thoroughly explained the data. The coding was iteratively developed across authors (led by LM and GL) and adjustments made where appropriate based on discussion. The analysis process showed that saturation had been reached as no new information was emerging from the later transcripts.

Findings

Participants:

Twenty people participated, with ages ranging from 18-74 (mean age 51), and 70% (N=14) were female. In addition approximately 50% (N=11) of participants had completed the trial at the time of interview. All patients had experienced at least two or three different treatment options for RTI either as part of the trial or in the past.

Themes:

Thematic analysis identified a total of five key themes relating to patients' views of different management approaches, and factors which may influence a patient's decision to use a self-help treatment (paracetamol/ibuprofen/steam inhalation) or consult with a GP for the treatment of a RTI. These are shown in Table 1.

Table 1: Themes identified in analysis

Themes	Sub-themes
**1. Perceptions of illness**	-Duration of symptoms<br>-Perceived signs of severity<br>-Quality of life impact
**2. Advice from others**	-Acceptance of GP advice<br>-Consideration of alternative advice
**3. Perceptions of antibiotics**	-Antibiotics have unpleasant side-effects<br>-Concerns and misunderstandings

For peer review only

		about resistance<br>-Antibiotics needed for specific cases
**4. Perceptions of self-help treatment**		-Treatment duration short and irregular<br>-Experience of effective treatment
	**Paracetamol and ibuprofen**	-Apprehension of combination medication<br>-General dislike of medication<br>-Concerns of medication side-effects<br>-Medication acceptable for pain relief
	**Steam inhalation as a limited technique**	-Only beneficial if not severe<br>-Provides short term relief<br>-Relieves but not cures
**5. Perceptions of delayed prescribing**		-Viewed as a positive option<br>-Acceptance of GP recommended method<br>-Confusion of delayed prescribing role.

In the following sections we summarise themes 1 and 2 for context before reporting in depth on themes 3-5, describing each in turn, using exemplary quotations for illustrative purposes (participant number is shown in parentheses). Findings are discussed in relation to the most prevalent and influential themes outlined by participants.

1 and 2: Perceptions of illness severity, and advice from others

Participants' views on the severity of their condition related to the evaluation of the duration of symptoms. Participants signalled that they were mostly unaware of the natural history, and specific symptoms (such as breathing difficulty) were often highlighted as indicators that their condition might be severe, resulting in the decision to consult.

> *"I think it was because it was - I could feel or could hear a creaking or croaking in the chest cavity…So that said to me, right we need to go and get something to deal with it."(PL7)*

For peer review only

Participants reported making decisions on how to treat their condition based on advice from both GPs and alternative professional and lay sources which included pharmacists, family members and the media.

> *"I do the Karvol inhalation, my patent remedy…. That's an old one from my mother, so it's a long, long time ago, but….I think that'd be the best treatment"(P03)*

3. Perceptions of antibiotics

A patient's decision on how to treat their condition was reported as being influenced by their perceptions of antibiotics, such as relating to beliefs about unpleasant side effects and concerns about resistance. Patients who described unpleasant side-effects associated with the use of antibiotics tended to report reluctance to take them, unless absolutely necessary.

> *"I don't like taking antibiotics because, after all, whatever it does to the system in relation to the bowel side of it, it destroys all the bacteria there anyhow so it's better to, if you can get away without taking them, the better"(PL08)*

In line with existing literature that identified people may believe the body to be resistant rather than the bacteria[16], many participants reported concerns about antibiotic resistance, which influenced their decision on how to best treat their condition and symptoms. Participants reported the belief that antibiotics were necessary in some specific situations and circumstances, such as patients with co-morbidities.

> *"Well in my own particular case … I almost feel that if I do go the doctor's with a nasty cough or some sort of … you know, nasal blockage or ear ache, I almost thought I ought to be given antibiotics as a matter of course because of my asthma" (PL7)*

4. Perceptions of self-help treatment

Participants' perceptions surrounding the potential benefits and limitations of self-help treatments for RTI influenced their decision to try these or visit their GP. They reported that in general self-help treatments were used for short periods of time at irregular intervals, in

For peer review only - http://bmjopen.bmj.com/site/about/guidelines.xhtml

contrast with the likely prescribed management approach offered by a GP to take pain relief at regular intervals.

> *"My GP did say that managed pain is better, so take it regularly, but I tend not to do that; perhaps have some in the morning and if I feel really ropey in the afternoon, but then definitely in the evening." (PL8)*

In general, participants reported a dislike for self-help treatments which involved taking medication. However, it is interesting to note that despite views of disliking these medications, participants were more likely to use them if the GP advised it, and would be less likely to try these without such advice. Interviewees reported concerns and worries relating to possible side effects which taking paracetamol or ibuprofen may cause.

> *"Yes, isn't there a problem with that [ibuprofen] in relation to blood? Yes, there's something I've heard about ibuprofen, that particular medication, which I didn't think was too good...I would not want to take it in that sense."(PL01)*

Participants reported being apprehensive about taking a combination of ibuprofen and paracetamol together. The combination of medication was construed by some as somehow 'risky' when compared to taking a single type of medication.

> *"Well I should have thought that was a bit of a lethal cocktail together but I don't know; if it was up to me I wouldn't want to do that."(PL08)*

Despite concerns relating to taking paracetamol or ibuprofen for the treatment of RTI, participants reported that they were happy to take these for the use of pain relief. This was a familiar usage and appeared to be considered as more acceptable than using them for an RTI. Most reported steam inhalation could relieve symptoms to a degree, however it was perceived as only providing short-term relief from symptoms and that it would not assist in curing the underlying condition. Therefore if symptoms were considered as serious, using steam inhalation was not viewed as a potential overall treatment option.

For peer review only

"I don't think that they actually cure me, but I think they give me a little bit of relief from congestion." (PL09)

5. Perceptions of delayed prescribing

Delayed prescribing was generally viewed as a positive technique and patients reported being happy to accept whichever method of delayed prescribing the GP recommended. None of the participants expressed disappointment at not receiving an antibiotic prescription for immediate use. Delayed prescribing was generally viewed by participants as a positive technique which could give them reassurance of having access to a prescription just in case. The main benefit was seen as the patient having some decision in their own treatment while preventing them from unnecessarily taking antibiotics.

"I don't mind delayed prescribing. I mean, I would rather have them and know that I've got them and, yes, if I don't need them, then I wouldn't take them, but I like the safety [of a delayed prescription], knowing that I've got them, because more often than not, it doesn't go away" (P05)

Although happy to accept delayed prescribing as a treatment option, many participants were confused as to the purpose of the technique. Some reported confusion because they had been told their condition was viral yet had still been issued a delayed prescription, indicating the rationale for delayed prescribing may need to be clearer.

"…either you have or haven't got an infection that either does or doesn't need treating. And if it needs treating it needs treating now, if not it doesn't" (PA7)

Some expressed the view that the technique might be driven by economic reasons.

"I am more and more wondering if medication is being withheld because of expenses…surgeries have to make a living, they have to work within a budget…" (PA3)

Discussion

For peer review only - http://bmjopen.bmj.com/site/about/guidelines.xhtml

Main findings

Overall, the findings suggest participants were aware of the limitations of antibiotics and do not wish to receive them every time they consult a GP for RTIs. There was some uncertainty about the rationale for delayed prescribing with a particular risk of mixed messages, whereby on the one hand some expressed the conflict between being told their condition did not warrant immediate treatment because it may not be a bacterial infection, whilst on the other hand it might at some point require antibiotic treatment. This is a complicated message for GPs to deliver and for patients to receive, interpret and understand. It may be that GPs should not discuss viruses during the consultation, and rather emphasise that most types of RTIs settle on their own without the need to antibiotics. Some participants suspected that delayed prescribing was primarily driven by economic decisions and the need to cut spending, but most participants viewed delayed prescriptions positively, suggesting they were happy to accept their GP's recommendation. That said, what cannot be emphasised enough is the clear need for GPs and their patients to negotiate the delayed disposal and to render transparent the rationale for a delay.

Participants expressed concerns over self-help that involved taking medication, particularly painkillers in combination for the treatment of RTI. When patients did take medication some indicated irregular use of them, which contrasts with a need for regular and continued dosing, sometimes when symptoms appear to have reduced. Just taking 'drugs' when they feel they need to is intuitively logical and may signal a need for clearer information that people could get better and feel better more quickly if they take medication as recommended. Indeed, it seemed overall, despite reservations, participants conveyed an overall stance of a willingness to accept advice to use painkillers if recommended by their GP. Some interviewees seemed to require authorisation from their GP to try over-the-counter remedies: this may link to a misconception that analgesics do not help for respiratory symptoms. Whilst steam inhalation was viewed as an acceptable treatment participants generally described this method as providing short-term relief from non-severe symptoms. The decision to consult a GP was initially influenced by patient perceptions of the natural history and duration, a variety of personal indicators, and beliefs that contrast with documented natural history indices of severity.

Comparison with existing literature

For peer review only

Participants reported being aware of the limitations of antibiotics and not expecting to receive them every time they went to the GP with a RTI which supports previous research, and often contrasts with GP perceptions of high levels of patient pressure to prescribe[8]. However, the self-selected nature of the sample interviewed is likely to mean they were more open to exploring alternatives to antibiotics[17]. There is still considerable room for patients to have a better understanding of the limited benefit from antibiotics, such a patient information leaflets, given the evidence that beliefs in the effectiveness of antibiotics are still strong[13,18]. There is also room to ensure potential misunderstandings are managed well when a no-prescribing disposal is selected. For example, whilst not common, some interview accounts suggested that not prescribing antibiotics can be understood (at least in part) to be a resource saving strategy rather than a clinically driven one. Tonkin-Crine et al.[19] also found that older adults may hold such a view. In addition, the perception of a delayed script as potentially contradictory to GP advice that an antibiotic prescription may not be required also needs to be tackled when a prescribing decision is negotiated. Patients reported high levels of concordance with GP advice across all treatment options, including all methods of delayed prescribing. Previous studies suggest high patient compliance/concordance with GP advice is strongly associated with doctor-patient agreement, which is facilitated by patient understanding of treatment benefits[20]. Data presented here suggested participants would have been happy to accept a delayed prescription: these perceptions were supported by the quantitative satisfaction data from the trial[13]. Previous research identified patient satisfaction with delayed prescribing[21], but also a subgroup of patients who had expected antibiotics and were disappointed when they did not receive a prescription for immediate use. The current study in contrast did not find a group of patients who were disappointed at not receiving antibiotics. Speculatively, this may, in part, be associated with societal changes and the extensive publicity about antibiotic resistance. However, this also may reflect selection bias since trial patients may represent a sub-group who are interested in research, have more awareness of issues surrounding antibiotic prescribing, and are more open to trying different approaches and therefore happier to accept new advice or techniques. Furthermore, since all participants were taking part in a trial it is likely that GPs provided a detailed explanation of their treatment decision, using structured advice sheets, which is unlikely to happen in routine practice [22].

Implications for clinical practice

For peer review only

Overall, participants reported delayed prescribing to be an acceptable treatment option. Previous research[23] has suggested that patient understanding of the rationale for a particular treatment can increase patient satisfaction. In this study delayed prescribing was delivered using structured advice sheets detailing the expected natural history; benefits/disbenefits of antibiotics; how long to delay; and safety netting/symptom advice. The findings suggest good concordance and high satisfaction should accompany a consistent and thorough explanation of the approach. Participants did not display a strong preference of any particular method of delayed prescribing, which suggests any approach is likely to be acceptable in practice, as long as a clear explanation is provided. .

Findings suggest participants were concerned about using painkillers as a treatment method for RTI. They reported concerns relating to potential hazards of combining analgesics, which links with existing evidence of low analgesic concordance rates in patients with RTI[24]. However, interviewees did report that they thought paracetamol and ibuprofen were, at least in isolation, acceptable treatments for pain relief. The results of the main trial suggest that advice to consume regular ibuprofen either alone or in combination may be associated with worse outcomes and therefore a preference for paracetamol would be the recommended approach in future[13]. This finding suggests that some analgesics can be viewed as feasible treatment options for patients, but education and explanation of the justification and possible benefits/dis-benefits for these medications need to be shared with patients, as well as emphasising the role of pharmacists in terms of offering information and support. Regarding steam inhalation, results from the PIPS trial[13]] showed no impact on symptoms and minor scalds in some individuals; combined with the findings presented in this paper (mostly short term help at best) and prior literature[25] means that until more robust data are available, advice to use steam cannot be justified. More frequent/prolonged use of steam might help, but this would need to be shown before a recommendation could be made.

This qualitative study shows how patients' decisions to consult a GP were often related to perceived indicators of illness severity. These findings are consistent with Leventhal's (1984) model of illness representations[26] which suggests that patients' beliefs relating to the cause, identity, duration and controllability of an illness are all likely to influence the way in which they respond to the condition (e.g. consulting a GP). The GRACE INTRO European study used this model to develop a patient booklet and findings indicated it was well received

For peer review only

by patients and gave them new information about illness duration and how to self care[19,27]. Other resources have also been developed to improve patient understanding of RTIs in primary care[28,29,30]. As most of the indicators that patients used to determine illness severity may not warrant a GP consultation, it suggests a need to review the clarity of patient information (e.g. providing clear examples of symptoms and illness duration that would ordinarily merit a visit to the GP, and those which are not a cause for worry). The promotion of this information within the community, as well as dealing with the common misconception that antibiotic medication may help coughs and colds[17] could help to reduce unnecessary GP consultations in the future.

Strengths and limitations

One of the key strengths of the study is that all patients interviewed had experienced at least two or three different treatment options for RTI either as part of the trial or in the past. This included antibiotics, delayed prescribing, paracetamol, ibuprofen, and steam inhalation. Therefore, the interviews provided opportunity for discussion of the various treatment options with a targeted information-rich sample. This strengthens the findings and allows stronger conclusions to be drawn.

However, the sample of participants who took part in the interviews may have limited the scope of the findings somewhat, as they had all participated in the PIPS trial. This may have led to a sample of participants who were particularly interested in research of this nature and may not have represented views held by 'typical' patients, particularly views on antibiotic resistance. Also, the participants had all consulted for an RTI and therefore may have different views than individuals who may not consult for such symptoms. Whilst having incomplete information on patient characteristics including information on people who declined participation is a limitation of this study, the research does provides novel and relevant findings.

Conclusion

The findings suggest that delayed prescribing appears to be an acceptable technique, as long as the method of delayed prescribing is described well to optimise patient understanding of the rationale. Increased patient education relating to the safety and justification of using paracetamol for the treatment of RTI is likely to increase the acceptability of analgesics. In

For peer review only - http://bmjopen.bmj.com/site/about/guidelines.xhtml

addition, enhanced approaches to sharing information with patients- relating to the natural history of illness, the appropriate signs and symptoms requiring GP advice, and those symptoms and signs which do not require urgent attention – could help reduce unnecessary consultations.

Acknowledgements

We are grateful to all the general practitioners and practice nurses and particularly the patients who gave their time to this study.

PIPS Investigators

University of Southampton: Paul Little, Ian Williamson, Mike Moore, Mark Mullee, Jo Kelly, Julie Hooper, Lisa McDermott, Geraldine Leydon, Ben Holdstock-Brown*, Amanda Nagle*, Jennifer White* (*medical students at the time); Samantha Hall, Patient and Public (PPI) representative.

Author contribution

GM Leydon led the qualitative work as part of the PIPs trial. P Little secured research funding/and acted as overall PI of the PIPs trial. J Kelly facilitated recruitment. L McDermott, A Nagle, and J White collected interview data. All authors were involved in/commented on data analysis (led by GM Leydon and L McDermott). A Halls and GM Leydon developed the manuscript, to which all authors contributed.

Funding statement

This article presents independent research funded by the National Institute for Health Research (NIHR) under its Programme Grants for Applied Research programme (grant ref No RP-PG-0407-10098). The views expressed are those of the author(s) and not necessarily those of the NHS, the NIHR or the department of Health.

Competing interests statement

There are no competing interests to report.

Ethical approval

For peer review only

The study was approved by Southampton and Southwest MREC number 06/Q1702/154 and all participants gave informed consent.

Data sharing

No additional data available.

References

1. Dagan R, Leibovitz E, Greenberg D, Yagupsky P, Fliss DM, Leiberman A. Dynamics of pneumococcal nasopharyngeal colonization during the first days of antibiotic treatment in pediatric patients. *J Ped Infect Dis* 1998; 17:880-5.
2. Costelloe C, Metcalfe C, Lovering A, Mant D, Hay A. Effect of antibiotic prescribing in primary care on antimicrobial resistance in individual patients: Systematic review and meta-analysis. *BMJ* 2010;340:c.2096.
3. World Health Organisation. The evolving threat of antimicrobial resistance: Options for action. WHO; 2012.
4. Little P, Watson L, Morgan S, Williamson I. Antibiotic prescribing and admissions with major suppurative complications of respiratory tract infections: a data linkage study. *Br J Gen Pract* 2002;52(476):187-90.
5. Macfarlane J, Holmes W, Gard P, Thornhill D, Macfarlane R, Hubbard R. Reducing antibiotic use for acute bronchitis in primary care: blinded, randomised controlled trial of patient information leaflet. *BMJ* 2002; 324:91-4.
6. NICE Guideline Development Group. Respiratory tract infections – antibiotic prescribing: Prescribing of antibiotics for self-limiting respiratory tract infections in adults and children in primary care. London: NICE; 2008
7. ESPAUR report Public Health England. English surveillance programme for antimicrobial utilisation and resistance (ESPAUR) 2010-2014. London: Public Health England; 2015.
8. Little P, Rumsby K. Kelly J, Watson L, Moore M, Warner G, et al. Information leaflet and antibiotic prescribing strategies for acute lower respiratory tract infection: a randomised controlled trial. *JAMA* 2005;293:3029-35.
9. De la Poza Abad MD et al: Prescription strategies in acute uncomplicated respiratory

For peer review only

infections: A randomized clinical trial. *JAMA Intern Med.* 2016;176(1):21-29. doi:10.1001/jamainternmed.2015.7088

10. Hollinghurst S, Redmond N, Costelloe C, Montgomery A, Fletcher M, Peters TJ, Hay AD. Paracetamol plus ibuprofen for the treatment of fever in children (PITCH). *BMJ* 2008;337:a1490.
11. Singh M, Heated, humidified air for the common cold. *Cochrane Library* 2004;2:CD001728.
12. Burke P, Bain J, Robinson, D, Dunleavey J. Acute red ear in children: controlled trial of non-antibiotic treatment in general practice. *BMJ* 1991;303:558-562
13. Little P, Moore M, Kelly J et al. Ibuprofen, paracetamol, and steam for patients with respiratory tract infections in primary care: pragmatic randomised factorial trial *BMJ* 2013;347:f6041.
14. Hammersley M: *What's Wrong With Ethnography?* London: Routledge, 1992.
15. Braun V, and Clarke V, Using thematic analysis in psychology. *Qual Res in Psych* 2006; 3:77-101.
16. Brookes-Howell L, Elwyn G, Hood K, Wood F, Cooper L, Goossens H, Ieven M, Butler CC. 'The body gets used to them': patients' interpretations of antibiotic resistance and the implications for containment strategies *J Gen Intern Med* 2012;27(7):766-72.
17. McNulty CAM, Nichols T, French DP, Joshi P, Butler CC. 'Expectations for consultations and antibiotics for respiratory tract infection in primary care: the RTI clinical iceberg *Brit J Gen Pract* 2013;63(612):e.429-e.436.
18. McNulty CAM, Boyle P, Nichols T, Clappison P, and Davey P. The public's attitudes to and compliance with antibiotics. *J Antimicrob Chemo* 2007; 60 (suppl 1):i63-i68.
19. Tonkin-Crine S, Anthierens S, et al. 'Exploring patients' views of primary care consultations with contrasting interventions for acute cough: a six-country European qualitative study, npj *Primary Care Respiratory Medicine* 2014;24:14026.
20. Kerse N, Buetow S, Mainous AG, Young, G, Coster G, Arroll B. 'Physician-patient relationship and medication compliance: A primary care investigation. *Ann Fam Med* 2004;2:455-461.
21. Arroll BA, Goodyear-Smith F, Thomas DR, Kerse N. Delayed antibiotic prescriptions: what are the experiences and attitudes of physicians and patients? *J Fam Pract* 2002;51;954-9.
22. Little P, Moore M, Kelly J, Williamson I, Leydon G, McDermott L, Mullee M, Stuart

For peer review only

B. Delayed antibiotic prescribing strategies for respiratory tract infections in primary care: pragmatic, factorial, randomised controlled trial. *BMJ* 2014; 14:348

23. Francis NA, Phillips R, Wood F, Hood K, Simpson S, Butler CC. Parents' and clinicians' views of an interactive booklet about respiratory tract infections in children: a qualitative process evaluation of the EQUIP randomised controlled trial. *BMC Family Practice* 2013;14:182.
24. Little P, Gould C, Williamson I, Moore M, Warner G, Dunleavey J. A pragmatic randomised controlled trial of two prescribing strategies for acute otitis media. BMJ 2001;332:336-42.
25. Singh M. Heated, humidified air for the common cold. Cochrane Library 2004;2:CD001728
26. Leventhal H, Nerenz DR, Steel DJ. Illness representations and coping with health threats. In: Baum A, Taylor SE, Singer JK, editors. Handbook of Psychology and Health: Social psychological aspects of health. Hillsdale, NJ: Earlbaum, 1984.
27. Yardley L, Douglas E, Athierens S, Tonkin-Crine S, O'Reilly G, Stuart B. et al. Evaluation of a web-based intervention to reduce antibiotic prescribing for LRTI in six European countries: quantitative process analysis of the GRACE/INTRO randomised controlled trial *Implementation Science* 2013;8:134
28. Francis N, Butler C, Hood K, Simpson S, Wood F, Nuttall J. Effect of using an interactive booklet about childhood respiratory tract infections in primary care consultations on reconsulting and antibiotic prescribing: a cluster randomised controlled trial *BMJ* 2009;339:b2885
29. TARGET Treating Your Infection patient leaflet version 5, available at http://www.rcgp.org.uk/clinical-and-research/toolkits/target-antibiotics-toolkit/patient-information-leaflets.aspx.
30. Cabral C, Ingram J, Lucas P. TARGET Children with Cough leaflet, available at http://child-cough.bristol.ac.uk.

For peer review only

Appendix 1

Introduction: Topics to be covered

The aim of this interview is to understand (1) the diagnosis and management of respiratory tract infections (RTIs) and your understanding of what has caused your infection (2) Patients' views on using paracetamol, ibuprofen and antibiotics.

If you could start at the beginning and tell me what led you/your child coming to see the doctor/nurse with your/your child's infection?

Prompts: Severity? Duration? Self-help- tried, if so what?

Previous experience of sore throat?
Previous experience of seeing doctor and getting treatment?
Family/friend/social support network?

We are interested in patient views on what causes RTI. Could you tell me what you think may have caused your illness or where you got it from?

Prompt: Bacteria? (If so, where from?) Virus? Weak system? Past/family history?

Could you please tell me if you have previously used paracetamol?

Frequency? Reason?

What are your views about taking paracetamol?

Prompt: Is taking full doses a problem or harmful in any way- e.g. is fever 'good'? Concerns?

Have you ever heard about problems with paracetamol?

e.g. poison; side effects

Do you remember the advice about paracetamol?

Prompt: Clear advice? Did you use it regularly rather than on demand? Did you use full doses?

If you stopped using it, why? (e.g. severity of symptoms settling, otherwise coping?)

Have you previously used ibuprofen? How do you feel about it?

Prompt: Is taking full doses a problem or harmful in any way- e.g. is fever 'good'?
Have you heard about problems with ibuprofen? (e.g. ulcers; side effects?)

For peer review only

Do you remember the advice about ibuprofen?
Did you use it regularly rather than on demand? Did you use full doses?
If you stopped using it, why? (e.g. severity of symptoms settling, otherwise coping?)

Have you previously used steam inhalation? How do you feel about it?

Prompt: offer an explanation if participant unsure.

What made you decide to use steam inhalation? Did you receive any advice?
How often did you use it? (e.g. regularly, sporadically).
If you stopped using it, why? (e.g. severity of symptoms settling, otherwise coping?)

If you haven't used steam inhalation, why? Is this something you would try in the future, if so why(not)?

Have you previously had antibiotics?
How did you feel about them?
Have you heard about problems with antibiotics (e.g. resistance; side effects?)

Do you remember the advice about antibiotics?
Were you advised to wait, if so, how long?
What was your experience of waiting?

Have you come across delayed prescribing before?
Prompt: Provide more information if participant unsure.

Outline the four common methods of delayed prescribing.

How did you feel about the method of delayed prescribing you were offered?
OR
How do you think you would feel about being offered delayed prescribing if you/your child consulted again?

If you started antibiotics, why? (e.g. severity of symptoms, general deterioration in well-being).

In your consultation with the doctor, how did you feel the doctor responded to your infection?"

Prompt: How did you feel about the consultation you had? Sympathetic? Enough information? Clear advice?

Were you given other self-help advice? If so, what did you think about it?

What other self-help measures did you use (e.g. tepid sponging?)

Conclusion

For peer review only

Are there any relevant issues we haven't covered that you would like to mention?

Are there any questions you would like to ask me?

For peer review only

COREQ (COnsolidated criteria for REporting Qualitative research) Checklist

A checklist of items that should be included in reports of qualitative research. You must report the page number in your manuscript where you consider each of the items listed in this checklist. If you have not included this information, either revise your manuscript accordingly before submitting or note N/A.

Topic	Item No.	Guide Questions/Description	Reported on Page No.
**Domain 1: Research team and reflexivity**			
Personal characteristics			
Interviewer/facilitator	1	Which author/s conducted the interview or focus group?	4
Credentials	2	What were the researcher's credentials? E.g. PhD, MD	4
Occupation	3	What was their occupation at the time of the study?	4
Gender	4	Was the researcher male or female?	4
Experience and training	5	What experience or training did the researcher have?	4
Relationship with participants			
Relationship established	6	Was a relationship established prior to study commencement?	N/A
Participant knowledge of the interviewer	7	What did the participants know about the researcher? e.g. personal goals, reasons for doing the research	N/A
Interviewer characteristics	8	What characteristics were reported about the inter viewer/facilitator? e.g. Bias, assumptions, reasons and interests in the research topic	N/A
**Domain 2: Study design**			
Theoretical framework			
Methodological orientation and Theory	9	What methodological orientation was stated to underpin the study? e.g. grounded theory, discourse analysis, ethnography, phenomenology, content analysis	4
Participant selection			
Sampling	10	How were participants selected? e.g. purposive, convenience, consecutive, snowball	3
Method of approach	11	How were participants approached? e.g. face-to-face, telephone, mail, email	3
Sample size	12	How many participants were in the study?	4
Non-participation	13	How many people refused to participate or dropped out? Reasons?	Unknown
Setting			
Setting of data collection	14	Where was the data collected? e.g. home, clinic, workplace	4
Presence of non-participants	15	Was anyone else present besides the participants and researchers?	No
Description of sample	16	What are the important characteristics of the sample? e.g. demographic data, date	4
Data collection			
Interview guide	17	Were questions, prompts, guides provided by the authors? Was it pilot tested?	4 and appendi
Repeat interviews	18	Were repeat inter views carried out? If yes, how many?	No
Audio/visual recording	19	Did the research use audio or visual recording to collect the data?	4
Field notes	20	Were field notes made during and/or after the inter view or focus group?	
Duration	21	What was the duration of the inter views or focus group?	3
Data saturation	22	Was data saturation discussed?	4
Transcripts returned	23	Were transcripts returned to participants for comment and/or	No

For peer review only

Topic	Item No.	Guide Questions/Description	Reported on Page No.
		correction?	
**Domain 3: analysis and findings**			
Data analysis			
Number of data coders	24	How many data coders coded the data?	4
Description of the coding tree	25	Did authors provide a description of the coding tree?	No
Derivation of themes	26	Were themes identified in advance or derived from the data?	4
Software	27	What software, if applicable, was used to manage the data?	N/A
Participant checking	28	Did participants provide feedback on the findings?	N/A
Reporting			
Quotations presented	29	Were participant quotations presented to illustrate the themes/findings? Was each quotation identified? e.g. participant number	5 onwards
Data and findings consistent	30	Was there consistency between the data presented and the findings?	5
Clarity of major themes	31	Were major themes clearly presented in the findings?	5
Clarity of minor themes	32	Is there a description of diverse cases or discussion of minor themes?	5

Developed from: Tong A, Sainsbury P, Craig J. Consolidated criteria for reporting qualitative research (COREQ): a 32-item checklist for interviews and focus groups. *International Journal for Quality in Health Care*. 2007. Volume 19, Number 6: pp. 349 – 357

Once you have completed this checklist, please save a copy and upload it as part of your submission. DO NOT include this checklist as part of the main manuscript document. It must be uploaded as a separate file.

For peer review only
